# Pervasive exposure of wild small mammals to legacy and currently used pesticide mixtures in arable landscapes

**DOI:** 10.1038/s41598-022-19959-y

**Published:** 2022-09-23

**Authors:** Clémentine Fritsch, Brice Appenzeller, Louisiane Burkart, Michael Coeurdassier, Renaud Scheifler, Francis Raoul, Vincent Driget, Thibaut Powolny, Candice Gagnaison, Dominique Rieffel, Eve Afonso, Anne-Claude Goydadin, Emilie M. Hardy, Paul Palazzi, Charline Schaeffer, Sabrina Gaba, Vincent Bretagnolle, Colette Bertrand, Céline Pelosi

**Affiliations:** 1 UMR 6249 Chrono-environnement, CNRS - Université de Franche-Comté, 16 Route de Gray, 25030 Besançon Cedex, France; 2 LTSER “Zone Atelier Arc Jurassien”, 25030 Besançon Cedex, France; 3 Department of Population Health, Luxembourg Institute of Health, 29 Rue Henri Koch, 4354 Esch-sur Alzette, Luxembourg; 4 UMR 7372 CEBC, CNRS-La Rochelle Université, USC INRAE, 405 Route de Prissé la Charrière, 79360 Villiers-en-Bois, France; 5 LTSER “Zone Atelier Plaine & Val De Sèvre”, 79360 Beauvoir Sur Niort, France; 6 UMR 1402 EcoSys, INRAE-AgroParisTech-Université Paris-Saclay, RD 10 Route de St Cyr, 78026 Versailles Cedex, France; 7 UMR EMMAH, INRAE-Avignon Université, 84000 Avignon, France

**Keywords:** Environmental chemistry, Agroecology, Conservation biology

## Abstract

Knowledge gaps regarding the potential role of pesticides in the loss of agricultural biodiversity worldwide and mixture-related issues hamper proper risk assessment of unintentional impacts of pesticides, rendering essential the monitoring of wildlife exposure to these compounds. Free-ranging mammal exposure to legacy (Banned and Restricted: BRPs) and currently used (CUPs) pesticides was investigated, testing the hypotheses of: (1) a background bioaccumulation for BRPs whereas a “hot-spot” pattern for CUPs, (2) different contamination profiles between carnivores and granivores/omnivores, and (3) the role of non-treated areas as refuges towards exposure to CUPs. *Apodemus* mice (omnivore) and *Crocidura* shrews (insectivore) were sampled over two French agricultural landscapes (*n* = 93). The concentrations of 140 parent chemicals and metabolites were screened in hair samples. A total of 112 compounds were detected, showing small mammal exposure to fungicides, herbicides and insecticides with 32 to 65 residues detected per individual (13–26 BRPs and 18–41 CUPs). Detection frequencies exceeded 75% of individuals for 13 BRPs and 25 CUPs. Concentrations above 10 ng/g were quantified for 7 BRPs and 29 CUPs (in 46% and 72% of individuals, respectively), and above 100 ng/g for 10 CUPs (in 22% of individuals). Contamination (number of compounds or concentrations) was overall higher in shrews than rodents and higher in animals captured in hedgerows and cereal crops than in grasslands, but did not differ significantly between conventional and organic farming. A general, ubiquitous contamination by legacy and current pesticides was shown, raising issues about exposure pathways and impacts on ecosystems. We propose a concept referred to as “biowidening”, depicting an increase of compound diversity at higher trophic levels. This work suggests that wildlife exposure to pesticide mixtures is a rule rather than an exception, highlighting the need for consideration of the exposome concept and questioning appropriateness of current risk assessment and mitigation processes.

## Introduction

The application of synthetic pesticides started by the late 1930s and exponentially grew after the World War II. In the 1960s, the publication of Rachel Carson’s book, along with some emblematic events of wildlife poisoning and the awareness of the global organochlorine pesticide contamination, has led to increased regulation rules of synthetic pesticides and the ban of many persistent organic pollutants (POPs)^1^. More recently, such concerns have still questioned the society and politics, leading to new enhanced regulation and monitoring (see for instance Rotterdam Convention http://chm.pops.int/^2,3^). Plant protection products (PPPs) are now among the chemicals with the strictest regulation, marketing and use authorizations of molecules being based on thorough Environmental Risk Assessment (ERA) and post-registration monitoring^2,4,5^. Currently used pesticides (CUPs) are designed and regulated in order to be safer (e.g. less persistent, less bioaccumulative, more targeted) than the former ones that have been banned or strictly restricted to specific situations (referred to as banned and restricted pesticides, BRPs, hereafter). The use of synthetic pesticides has increased over the past four decades in terms of total amount, diversity of molecules, and geographic expansion^6^. Nowadays, more than 500 active substances provided in several thousands of commercial products, belonging to more than 100 chemical classes with various modes of action are used worldwide^6^. Despite national or federal plans aiming at reducing the use of pesticides^2,5,7^, this trend is not expected to be reversed. According to projections for 2100, a tenfold increase in PPP use is even expected, in relation to climate change and growth of human population^8^.

BRPs and their breakdown products are still present in the environment due to legacy contamination and long persistence, and they can be remobilized due to current practices on arable soils^9^. Their impact on biodiversity and the environment may thus persist long after they have been banned for use^6^. Although CUPs are overall less persistent and bioaccumulative than BRPs, several approved PPPs in Europe still pose chronic risk for reproduction and/or are classified as endocrine disruptors. Around 50 compounds meet two criteria of the “*persistent, bioaccumulative and toxic*” class of substances^10^. Moreover, recent studies showed accumulation of several CUPs in soils of various habitats within the agricultural landscapes^11^. Recent large scale surveys across Europe and worldwide monitoring studies of multi-class PPPs in arable soils showed a high occurrence of residues of both BRPs and CUPs^12,13^.

Despite a growing body of research showing that synthetic pesticides are important drivers of a global severe decline of wildlife and widespread loss of farmland biodiversity, crucial gaps in knowledge about how they impact ecological processes hamper our ability to understand, predict and mitigate their unintentional effects^6,14^. A critical step is to characterize the exposure of non-target species to these compounds under realistic conditions, but information about the contamination of wildlife by CUPs is lacking. For instance, most of the monitoring schemes on raptors consider POPs, especially organochlorine insecticides, or only specific pesticides involved in poisoning events such as anticholinesterases and anticoagulants^15^. Direct bird poisoning by CUPs or transfer in tri-trophic food webs have been addressed in a few studies, but only focused on specific compound classes such as neonicotinoids^16,17^. The rare studies dealing with CUPs in free-living fauna have however shown the potential for non-target wildlife to be exposed and even to bioaccumulate CUPs, highlighting the relevance and need for further research and data about this timely issue^18,19^.

Small mammals hold a central position in ERA procedure for the registration of PPPs as standard toxicity tests and toxicological data for human and mammals are determined on the basis of animal testing mostly done on laboratory rats and mice^20,21^. Small mammals are largely represented in the list of “mammalian indicator species”, “generic focal species” in the first tiers of ERA. Several wild species, such as the wood mouse *Apodemus sylvaticus* and the common shrew *Sorex araneus*, are identified as “focal species” in the last tier^22^. Rodents and insectivores are also of particular focus in post-registration surveys to assess impacts on mammals^22^. However, exposure and response data are mostly obtained from controlled laboratory experiments and modelling studies, based on single compound design approaches, and without long-term analyses of residue accumulation. Moreover, field surveys of small mammals are usually performed without any monitoring of bioaccumulation. Actual measurements of small mammal exposure to PPPs under field realistic conditions are therefore noticeably rare^23–26^. Yet, small mammals have a major functional role in terrestrial ecosystems and several species are considered as beneficial organisms in agro-ecosystems through predation over weed seeds and invertebrates^27–33^. Moreover, as abundant and widespread preys for numerous vertebrates, they are involved in the transfer of pollutants in food webs and in secondary poisoning of predators^34,35^. Small mammals are also involved in numerical responses and cascading effects on predator’s population by decreasing food resource for predators when prey populations are reduced^36,37^. Numerous rodent and shrew taxa are threatened worldwide^38^, and negative effects on populations related to pesticides may also be of conservation concern.

Nowadays, the studies addressing the survey of CUP residues in wildlife are mostly based on modelling studies (see for instance^39–41^) and on analytical measurements in tissues, the latest thus relying on sacrifice of animals or on necropsies of dead individuals collected via networks of epidemiological/toxicological surveillance. The ethical and scientific questions related to animal welfare in research has became during the last decades an integral part of regulation in many countries and an emergent topic with regards to the development of new methods in biology, ecology and ecotoxicology^42^. In this context keratinized tissues represent a promising matrix to survey wildlife exposure to chemicals. Indeed, metallic and organic contaminants have been successfully monitored in bird feathers, and trace metals have been measured in hair samples of wild small mammals^42–44^. Analyse of pesticides in human hair to characterize exposure have received an increasing interest since the 2010’s and is considered as a more relevant biomarker to assess chronic exposure than biological fluids because of the extended window of detection accessible with this matrix^45,46^. It is admitted that chemicals are mainly incorporated into hair bulb living cells from blood stream, thus molecule concentrations in hair are considered as representative of the internal dose during the time of hair sample growth^47^. Hair analysis has been shown as a successful tool to assess chronic exposure to pesticides in rats since, based on controlled exposure during several months to a mixture of pesticides belonging to various chemical families, concentrations of chemicals in hair were significantly correlated with exposure intensity and concentrations in plasma^45,48^.

The present study aimed at assessing the “real world” exposure profile of wild small mammals to pesticides, based on the measurement of residues in hairs. To better characterize the drivers of exposure, we compared residue bioaccumulation in cultivated and semi-natural habitats that are treated with PPPs (conventional cereal crops and grasslands) or non-treated (organic cereal crops and grasslands, and hedgerows) in two regions of France. The two sampling sites belong to the Long-Term Socio-Ecological Research (LTSER) network: the Zone Atelier Arc Jurassien (ZAAJ, https://zaaj.univ-fcomte.fr/) in North-Eastern France, and the Zone Atelier Plaine & Val de Sèvre (ZAPVS, https://za-plaineetvaldesevre.com/) in mid-Western France. We considered small mammal species widely distributed and abundant in agro-ecosystems in France, and having various trophic traits: wild mice *Apodemus sylvaticus and flavicollis,* granivorous/omnivorous rodents, and the greater whiter-toothed shrew *Crocidura russula*, an insectivorous small mammal. Owing to the pervasiveness of environmental contamination by BRPs, we expected a “general background contamination”, i.e. a high frequency of residue detection within populations whatever was the studied zone, the type of farming (conventional farming "CF" or organic farming "OF") or the habitat type, but at low concentrations in animals. Conversely, for CUPs, we hypothesized that some “hot-spots” of exposure occurred in treated habitats, with some small mammals exhibiting high concentrations, while low concentrations and low frequency of detection were expected in non-treated areas since CUPs are not supposed to persist and bioaccumulate. We also expected differences in profiles of exposure between the two species due to their different trophic level. Widely used insecticides such as neonicotinoids being systemic, rodents should exhibit higher levels than shrews because insecticide residues may be present in vegetation organs they feed on^49^. Further, since rodents might consume treated seeds, a greater exposure of mice than of shrews to substances used as seed dressing like triazoles and neonicotinoids was anticipated^23,50^. Preys of shrews such as earthworms can accumulate fungicides and insecticides, as well as herbicides like pendimethalin and diflufenican at high concentrations^11^. A trophic transfer to shrews could therefore result in higher levels of herbicides in this species. Most of the banned pesticides are known as persistent, lipophilic, bioaccumulative compounds that have the potential to biomagnify in food webs. Shrews feeding at a higher trophic level than rodents, they are expected to exhibit greater contamination by BRPs.

## Results

### Occurrence of pesticides in small mammals: general patterns

A total of 112 different compounds were detected over the 140 parent pesticides and metabolites screened in hair samples (80% of the compounds screened). The full lists of compounds with their acronyms, the details of their full names and chemical families are provided in Tables 1 and 2.

**Table 1:** Concentrations of banned and restricted pesticides (BRPs) in small mammal hair samples, classified by decreasing number of detection.

Rank	Compounds^a^	LOQ	LOD	Number of detections		Shrews *Crocidura russula*			Wild mice *Apodemus sylvaticus/flavicollis*			Chemical family	Type of molecule	Use	Year of ban
		(ng/g)		*n*	(%)	Min	Med	Max	Min	Med	Max				
1	DMP	20	0.171	93	(100)	0.171	4.92	**118**	0.331	3.28	**32.5**	OP	*M OP*	I	
2	PNP	1	3.092	93	(100)	3.09	5.76	**23.6**	5.43	8.11	**29.8**	OP	*M parathion*	I	2002
3	3,4-DCPU	5	0.224	93	(100)	0.224	1.24	**12.1**	0.471	4.38	**47.2**	Urea	*M Urea*	H	
4	DEP	5	0.383	93	(100)	0.526	1.52	**10.4**	0.383	0.757	**13.0**	OP	*M OP*	I	
5	PCP	1	0.153	93	(100)	0.169	0.385	2.21	0.153	0.505	**13.6**	OC	*M chlordane*	I/H	1988
6	3Me4NP	0.5	0.349	93	(100)	0.355	0.925	5.89	0.349	0.766	**11.7**	OP	*M fenitrothion*	I/B	2007
7	DCPMU	0.5	0.012	93	(100)	0.017	0.060	0.473	0.012	0.023	0.133	Urea	*M Urea*	H	
8	DETP	0.1	0.011	93	(100)	0.011	0.027	0.223	0.015	0.053	7.54	OP	*M OP*	I	
9	Fipronil	0.1	0.003	93	(100)	0.003	0.011	0.445	0.004	0.013	0.066	Phenylpyrazole	*P*	I	2005
10	Fipronil sulfone	0.5	0.028	89	(96)	0.030	0.264	6.08	< LD	0.057	0.271	Phenylpyrazole	*M fipronil*	I	2005
11	Trifluralin	0.1	0.001	88	(95)	< LD	0.009	0.087	< LD	0.010	0.092	Dinitroaniline	*P*	H	2008
12	DMTP	0.5	0.001	85	(91)	< LD	0.022	0.259	< LD	0.065	4.82	OP	*M OP*	I	
13	HCB	0	0.001	78	(84)	< LD	0.081	1.40	< LD	0.041	0.194	OC	*M chlordane*	F	1988
14	γ-HCH (lindane)	1	0.007	63	(68)	< LD	0.056	0.228	< LD	0.039	0.144	OC	*P*	I	1998
15	Terbutryn	0	0.023	62	(67)	< LD	0.103	0.464	< LD	< LD	0.346	Tria-zine/zinone	P/M*	H	2002
16	Fenuron	1	0.001	50	(54)	< LD	0.004	0.338	< LD	< LD	0.141	Urea	*P*	H	2007
17	DMST	0.5	0.017	40	(43)	< LD	0.092	3.54	< LD	< LD	0.368	Amide pesticide	*M tolylfluanide*	F	2007
18	Flusilazole	0.5	0.001	34	(37)	< LD	< LD	0.107	< LD	< LD	0.005	Azole	*P*	F	2008
19	α-endosulfan	0.5	0.002	28	(30)	< LD	< LD	0.111	< LD	< LD	0.010	OC	*P*	I	2006
20	DMDTP	2	0.048	24	(26)	< LD	< LD	3.26	< LD	< LD	5.36	OP	*M OP*	I	
21	Diuron	0.5	0.020	23	(25)	< LD	< LD	0.481	< LD	< LD	< LD	Urea	*P*	H	2008
22	oxy-chlordane	1	0.010	16	(17)	< LD	< LD	0.074	< LD	< LD	0.010	OC	*M chlordane*	I	1972
23	trans-chlordane	0.5	0.001	13	(14)	< LD	< LD	0.186	< LD	< LD	0.169	OC	*P*	I	1972
24	Atrazine desethyl	1	0.074	12	(13)	< LD	< LD	1.04	< LD	< LD	0.234	Tria-zine/zinone	*M atrazine*	H	2001
25	Propoxur	1	0.036	9	(10)	< LD	< LD	0.175	< LD	< LD	0.134	Carbamate	*P*	I/B	2010
26	3,4-dichloroaniline	10	0.021	8	(9)	< LD	< LD	4.55	< LD	< LD	0.122	Urea	*M Urea*	H	
27	DEDTP	10	0.272	6	(6)	< LD	< LD	0.538	< LD	< LD	**23.0**	OP	*M OP*	I	
28	β-HCH	5	0.016	6	(6)	< LD	< LD	0.623	< LD	< LD	< LD	OC	*M lindane*	I	1998
29	o,p'-DDE	0.5	0.020	5	(5)	< LD	< LD	0.115	< LD	< LD	0.053	OC	*M DDT*	I	1987
30	α-HCH	0.5	0.012	5	(5)	< LD	< LD	0.028	< LD	< LD	< LD	OC	*M lindane*	I	1998
31	ε-HCH	0.5	0.033	5	(5)	< LD	< LD	0.291	< LD	< LD	< LD	OC	*M lindane*	I	1998
32	Malathion CA	0.5	0.128	4	(4)	< LD	< LD	0.128	< LD	< LD	2.52	OP	*M malathion*	I/B	2007
33	Atrazine	0.5	0.227	3	(3)	< LD	< LD	1.24	< LD	< LD	< LD	Tria-zine/zinone	*P*	H	2001
34	Methabenzthiazuron	0.5	0.027	3	(3)	< LD	< LD	0.097	< LD	< LD	0.027	Urea	*P*	H	2006
35	Carbaryl	0.5	0.029	3	(3)	< LD	< LD	0.074	< LD	< LD	< LD	Carbamate	*P*	I	2007
36	cis-chlordane	0.5	0.041	3	(3)	< LD	< LD	0.207	< LD	< LD	< LD	OC	*P*	I	1972
37	Simazine	0.5	0.030	2	(2)	< LD	< LD	0.063	< LD	< LD	< LD	Tria-zine/zinone	*P*	H	2001
38	Metoxuron	5	0.069	2	(2)	< LD	< LD	< LD	< LD	< LD	0.153	Urea	*P*	H	2006
39	Methomyl	0.5	0.002	2	(2)	< LD	< LD	< LD	< LD	< LD	0.002	Carbamate	*P*	I	2008
40	Dieldrin	0.5	0.050	2	(2)	< LD	< LD	0.153	< LD	< LD	< LD	OC	*P*	I	1972
41	Heptachlor-exo-epoxide	0.5	0.077	2	(2)	< LD	< LD	0.096	< LD	< LD	< LD	OC	*M heptachlor*	I	1972
42	IMPy (diazinon)	0.5	0.013	2	(2)	< LD	< LD	0.247	< LD	< LD	< LD	OP	*P*	I	2007
43	Fenarimol	1	0.001	1	(1)	< LD	< LD	< LD	< LD	< LD	0.001	Pyrimidine	*P*	F	2007
44	Prometryn	0.5	0.043	1	(1)	< LD	< LD	0.043	< LD	< LD	< LD	Tria-zine/zinone	*P*	H	2007
45	Sebuthylazine	0.5	0.079	1	(1)	< LD	< LD	0.079	< LD	< LD	< LD	Tria-zine/zinone	*P*	H	2004
46	Terbuthylazine	0.5	0.102	1	(1)	< LD	< LD	0.102	< LD	< LD	< LD	Tria-zine/zinone	*P*	H	2001
47	Chloroxuron	1	0.150	1	(1)	< LD	< LD	0.150	< LD	< LD	< LD	Urea	*P*	H	2007
48	Carbofuran	0.5	0.010	1	(1)	< LD	< LD	0.010	< LD	< LD	< LD	Carbamate	*P*	I	2008
49	Heptachlor	0.1	0.004	1	(1)	< LD	< LD	0.004	< LD	< LD	< LD	OC	*P*	I	1972
50	Heptachlor-endo-epoxide	1	0.076	1	(1)	< LD	< LD	0.076	< LD	< LD	< LD	OC	*M heptachlor*	I	1972
51	p,p'-DDE	2	1.678	1	(1)	< LD	< LD	1.678	< LD	< LD	< LD	OC	*M DDT*	I	1987
52	Bitertanol	2	nd	0		< LD	< LD	< LD	< LD	< LD	< LD	Azole	*P*	F	2010
53	Alachlor	1	nd	0		< LD	< LD	< LD	< LD	< LD	< LD	Amide pesticide	*P*	H	2006
54	Propazine	0.5	nd	0		< LD	< LD	< LD	< LD	< LD	< LD	Tria-zine/zinone	*P*	H	2007
55	Monolinuron	0.5	nd	0		< LD	< LD	< LD	< LD	< LD	< LD	Urea	*P*	H	2003
56	Promecarb	0.5	nd	0		< LD	< LD	< LD	< LD	< LD	< LD	Carbamate	*P*	I	2007
57	Propargite	5	nd	0		< LD	< LD	< LD	< LD	< LD	< LD	Sulfone sulfonate	*P*	I	2010
58	Aldrin	1	nd	0		< LD	< LD	< LD	< LD	< LD	< LD	OC	*P*	I	1972
59	Endrin	0.5	nd	0		< LD	< LD	< LD	< LD	< LD	< LD	OC	*P*	I/R	1991
60	Isodrin	2	nd	0		< LD	< LD	< LD	< LD	< LD	< LD	OC	*P*	I	1991
61	o,p'-DDD	2	nd	0		< LD	< LD	< LD	< LD	< LD	< LD	OC	*M DDT*	I	1987
62	p,p'-DDD	5	nd	0		< LD	< LD	< LD	< LD	< LD	< LD	OC	*M DDT*	I	1987
63	o,p'-DDT	5	nd	0		< LD	< LD	< LD	< LD	< LD	< LD	OC	*P*	I	1987
64	p,p'-DDT	2	nd	0		< LD	< LD	< LD	< LD	< LD	< LD	OC	*P*	I	1987
65	β-endosulfan	0.5	nd	0		< LD	< LD	< LD	< LD	< LD	< LD	OC	*P*	I	2006
66	δ-HCH	1	nd	0		< LD	< LD	< LD	< LD	< LD	< LD	OC	*M lindane*	I	1998
67	Crimidine	1	nd	0		< LD	< LD	< LD	< LD	< LD	< LD	Pyrimidine	*P*	R	2004

Maximum concentrations higher than 10 ng/g are in bold. The year of ban of BRPs is indicated for France when available, otherwise for European Union; note that effective use in the field might have ceased a few months/years before or after the vote of the regulation; the year is also indicated for specific metabolites as the year of ban of the parent compound. Abbreviations in headers: *LOQ* limit of quantification, *LD* Lowest detected value (nd, not detected), *Min* minimum, *Med* median, *Max* maximum. Abbreviations for chemical family and type of molecule: *OP* Organophosphorous, *OC* Organochlorine, *P* parent chemical, *M* metabolite. Abbreviations for type of use: *B* biocide, *F* fungicide, *H* herbicide, *I* insecticide, *R* rodenticide.

*Also a metabolite of triazines.

^a^List of compound abbreviations: 3,4-DCPU: 1-(3,4-Dichlorophenyl)urea; DCPMU : 1-(3,4-Dichlorophenyl)-3-methylurea; (o,p’ and p,p’)-DDD: (o,p’ and p,p’)-dichlorodiphenyldichloroethane; (o,p’ and p,p’)-DDE: (o,p’ and p,p’)-dichlorodiphenyldichloroethylene; (o,p’ and p,p’)-DDT: (o,p’ and p,p’)-dichlorodiphenyltrichloroethane; DEP: diethylphosphate; DETP: diethylthiophosphate; DEDTP: diethyldithiophosphate; DMP: dimethylphosphate; DMDTP: dimethyldithiophosphate; DMTP: dimethylthiophosphate; DMST: dimethylsulftoluidide; HCB: hexachlorobenzene; (α, β, γ, δ, ε)-HCH: (α, β, γ, δ, ε)-hexachlorocyclohexane; IMPy: 2-isopropyl-4-méthyl-6-hydroxypyrimidine; Malathion CA: malathion under form of carboxylic acid; 3Me4NP: 3-méthyl-4-nitrophenol; PCP: pentachlorophenol; PNP: p-nitrophenol.

**Table 2:** Concentrations of currently used pesticides (CUPs) in small mammal hair samples, ordered by decreasing number of detection.

Rank	Compounds^a^	LOQ	LOD	Number of detections		Shrews *Crocidura russula*			Wild mice *Apodemus sylvaticus/flavicollis*			Chemical family	Type of molecule	Use
		(ng/g)		*n*	(%)	Min	Med	Max	Min	Med	Max			
1	MCPA	2	2.275	93	(100)	2.28	4.81	**428**	2.47	3.88	**159**	Acid herbicide	*P*	H
2	Carbendazim	0.5	0.377	93	(100)	0.385	1.13	**200**	0.377	0.785	**172**	Carbamate	*P*	F
3	Prosulfocarb	1	0.535	93	(100)	0.535	1.29	**11.3**	0.705	1.14	**20.7**	Thiocarbamate	*P*	H
4	S-Metolachlor	0.03	0.009	93	(100)	0.018	0.059	**121**	0.009	0.073	**21.7**	Chloroacetanilid	*P*	H
5	3-PBA	0.5	0.100	93	(100)	0.236	1.11	**19.6**	0.100	0.344	**10.8**	Pyrethroid	*M Pyrethroid*	I/B
6	Azoxystrobin	0.5	0.013	93	(100)	0.046	0.207	**18.9**	0.013	0.049	1.66	Strobilurin	*P*	F
7	2,4-D	0.1	0.045	93	(100)	0.208	0.915	**34.1**	0.045	0.219	4.56	Acid herbicide	*P*	H
8	Cl_2_CA	0.5	0.072	93	(100)	0.152	0.966	**16.7**	0.072	0.243	7.02	Pyrethroid	*M Pyrethroid*	I/B
9	Dimethachlor	0.5	0.001	93	(100)	0.001	0.004	0.019	0.003	0.039	0.271	Amide pesticide	*P*	H
10	TCPy	0.3	0.153	93	(100)	0.266	0.724	3.53	0.153	0.362	1.39	OP	*M chlorpyrifos*	I
11	Dichlorprop	1	0.022	92	(99)	< LD	0.482	**500**	0.022	0.161	**11.8**	Acid herbicide	*P*	H
12	Lenacil	1	0.200	92	(99)	< LD	0.266	0.995	0.222	0.360	0.917	Uracil	*P*	H
13	Pendimethalin	2	0.157	87	(94)	< LD	0.514	2.20	< LD	0.393	5.44	Dinitroaniline	*P*	H
14	Thiacloprid	5	0.013	83	(89)	0.014	0.249	2.67	< LD	0.036	2.86	Neonicotinoid	*P*	I
15	Metazachlor	0.5	0.010	82	(88)	< LD	0.023	0.298	< LD	0.019	0.093	Chloroacetanilid	*P*	H
16	Imidacloprid	0.5	0.053	80	(86)	0.160	8.37	**70.7**	< LD	0.129	0.829	Neonicotinoid	*P*	I
17	Tebuconazole	1	0.003	80	(86)	< LD	0.780	**74.4**	< LD	0.109	**42.5**	Azole	*P*	F
18	Epoxiconazole	0.5	0.155	79	(85)	0.155	1.94	**162**	< LD	0.329	**35.9**	Azole	*P*	F
19	ClCF_3_CA	5	0.008	79	(85)	< LD	0.066	0.940	< LD	0.024	0.389	Pyrethroid	*M Pyrethroid*	I/B
20	Mecoprop	1	0.057	78	(84)	< LD	0.684	**133**	< LD	0.093	**13.9**	Acid herbicide	*P*	H
21	Boscalid	2	0.014	75	(81)	< LD	1.086	**355**	< LD	0.116	**146**	Carboxamide	*P*	F
22	Thiabendazole	0.5	0.008	75	(81)	< LD	0.053	0.896	< LD	0.022	0.114	Azole	*P*	F
23	Pyraclostrobin	0.5	0.001	73	(78)	< LD	0.055	**32.8**	< LD	0.005	**18.7**	Strobilurin	*P*	F
24	Prochloraz	0.5	0.003	73	(78)	< LD	0.107	**22.0**	< LD	0.001	**341**	Azole	*P*	F
25	Propiconazole	0.5	0.039	72	(77)	< LD	0.618	**92.6**	< LD	0.048	**30.8**	Azole	*P*	F
26	Cyproconazole	0.5	0.004	68	(73)	< LD	0.320	**77.8**	< LD	< LD	3.17	Azole	*P*	F
27	Isoproturon	2	0.008	64	(69)	< LD	0.143	**73.5**	< LD	< LD	0.195	Urea	*P*	H
28	Propyzamide	1	0.010	60	(65)	< LD	0.320	**270**	< LD	< LD	**32.1**	Benzamide	*P*	H
29	Chlortoluron	1	0.036	54	(58)	< LD	0.123	**34.5**	< LD	< LD	**11.0**	Urea	*P*	H
30	Cypermethrine	2	0.049	51	(55)	< LD	0.087	1.34	< LD	0.137	6.38	Pyrethroid	*P*	I/B
31	Oxadiazon	0.5	0.001	49	(53)	< LD	0.010	0.054	< LD	< LD	0.126	Oxadiazin	*P*	H
32	Trifloxystrobin	0.5	0.002	47	(51)	< LD	0.004	1.33	< LD	< LD	3.82	Strobilurin	*P*	F
33	Diflufenican	0.5	0.055	45	(48)	< LD	0.157	**168**	< LD	< LD	1.76	Carboxamide	*P*	H
34	Zoxamide	0.5	0.007	39	(42)	< LD	0.016	0.145	< LD	< LD	0.040	Benzamide	*P*	F
35	Difenoconazole	0.5	0.005	27	(29)	< LD	< LD	0.157	< LD	< LD	0.174	Azole	*P*	F
36	Cyhalothrine	0.5	0.145	25	(27)	< LD	< LD	2.27	< LD	< LD	**13.4**	Pyrethroid	*P*	I/B
37	2,4-DB	0.5	0.039	24	(26)	< LD	< LD	3.45	< LD	< LD	9.09	Acid herbicide	*P*	H
38	Br_2_CA	0.5	0.028	23	(25)	< LD	< LD	2.18	< LD	< LD	10.8	Pyrethroid	*M Pyrethroid*	I/B
39	Tetraconazole	1	0.013	20	(22)	< LD	< LD	14.1	< LD	< LD	< LD	Azole	*P*	F
40	4F3PBA	0.5	0.009	19	(20)	< LD	< LD	0.160	< LD	< LD	6.36	Pyrethroid	*M Pyrethroid*	I/B
41	Acetamiprid	5	0.001	18	(19)	< LD	< LD	0.029	< LD	< LD	< LD	Neonicotinoid	*P*	I
42	MCPB	0.5	0.022	17	(18)	< LD	< LD	0.154	< LD	< LD	8.82	Acid herbicide	*P*	H
43	Permethrine	10	0.041	16	(17)	< LD	< LD	5.38	< LD	< LD	**14.2**	Pyrethroid	*P*	I/B
44	Cyprodinil	1	0.027	13	(14)	< LD	< LD	**14.6**	< LD	< LD	3.73	Anilino-pyrimidine	*P*	F
45	2-ClBA	0.5	0.013	13	(14)	< LD	< LD	0.180	< LD	< LD	5.36	Pyrethroid	*M Pyrethroid*	I/B
46	Cyfluthrine	2	0.066	12	(13)	< LD	< LD	0.726	< LD	< LD	**11.2**	Pyrethroid	*P*	I/B
47	Clothianidin	1	0.053	10	(11)	< LD	< LD	1.14	< LD	< LD	0.056	Neonicotinoid	P/M*	I
48	Aclonifen	50	0.400	9	(10)	< LD	< LD	**95.3**	< LD	< LD	**26.4**	Diphenyl-ether	*P*	H
49	Chloridazon	0.5	0.041	8	(9)	< LD	< LD	0.766	< LD	< LD	< LD	Triazine/Triazinone	*P*	H
50	Deltamethrine	2	0.732	5	(5)	< LD	< LD	0.781	< LD	< LD	6.61	Pyrethroid	*P*	I/B
51	Fenoxycarb	2	0.054	4	(4)	< LD	< LD	0.217	< LD	< LD	0.131	Carbamate	*P*	I
52	Spinosyn A	0.4	0.002	4	(4)	< LD	< LD	0.016	< LD	< LD	< LD	Macrolide	*P*	I
53	Fenvalerate	1.5	0.549	4	(4)	< LD	< LD	< LD	< LD	< LD	4.38	Pyrethroid	*P*	I/B
54	Pyrimethanil	0.5	0.078	3	(3)	< LD	< LD	< LD	< LD	< LD	0.160	Anilino-pyrimidine	*P*	F
55	Linuron	0.5	0.004	3	(3)	< LD	< LD	0.365	< LD	< LD	< LD	Urea	*P*	H
56	Fenbuconazole	1	0.007	2	(2)	< LD	< LD	0.012	< LD	< LD	< LD	Azole	*P*	F
57	Metamitron	5	0.196	2	(2)	< LD	< LD	5.24	< LD	< LD	< LD	Triazine/Triazinone	*P*	H
58	Oxamyl	0.5	0.055	2	(2)	< LD	< LD	0.814	< LD	< LD	< LD	Carbamate	*P*	I
59	Bifenthrine	0.5	1.332	2	(2)	< LD	< LD	< LD	< LD	< LD	4.68	Pyrethroid	*P*	I/B
60	Penconazole	1	0.013	1	(1)	< LD	< LD	0.013	< LD	< LD	< LD	Azole	*P*	F
61	Iprodione	5	0.095	1	(1)	< LD	< LD	< LD	< LD	< LD	0.095	Dicarboximide	*P*	F
62	Imazalil	10	nd	0		< LD	< LD	< LD	< LD	< LD	< LD	Azole	*P*	F
63	Myclobutanil	0.5	nd	0		< LD	< LD	< LD	< LD	< LD	< LD	Azole	*P*	F
64	Triadimenol	20	nd	0		< LD	< LD	< LD	< LD	< LD	< LD	Azole	*P*	F
65	Iprovalicarb	0.5	nd	0		< LD	< LD	< LD	< LD	< LD	< LD	Carbamate	*P*	F
66	Fenhexamid	10	nd	0		< LD	< LD	< LD	< LD	< LD	< LD	Amide pesticide	*P*	F
67	Kresoxim-methyl	0.5	nd	0		< LD	< LD	< LD	< LD	< LD	< LD	Strobilurin	*P*	F
68	Metribuzin	20	nd	0		< LD	< LD	< LD	< LD	< LD	< LD	Triazine/Triazinone	*P*	H
69	Metobromuron	0.5	nd	0		< LD	< LD	< LD	< LD	< LD	< LD	Urea	*P*	H
70	Dinotefuran	2	nd	0		< LD	< LD	< LD	< LD	< LD	< LD	Neonicotinoid	*P*	I
71	Thiamethoxam	0.4	nd	0		< LD	< LD	< LD	< LD	< LD	< LD	Neonicotinoid	*P*	I
72	Indoxacarb	0.5	nd	0		< LD	< LD	< LD	< LD	< LD	< LD	Oxadiazin	*P*	I
73	Dimethoate	0.5	nd	0		< LD	< LD	< LD	< LD	< LD	< LD	OP	*P*	I

Maximum concentrations higher than 10 ng/g are in bold. Abbreviations in headers and values: *LOQ*, limit of quantification; *LD*, Lowest detected value (*nd*, not detected); *Min*, minimum; *Med*, median; *Max*, maximum. Abbreviations for chemical family and type of molecule: *OP*, Organophosphorous; *OC*, Organochlorine; *P*, parent molecule; *M*, metabolite. Abbreviations for type of use: *B*, biocide; *F*, fungicide; *H*, herbicide; *I*, insecticide; *R*, rodenticide.

*Also a metabolite of Thiametoxam.

^a^List of abbreviations: Br_2_CA: acid 3-(2,2-dibromo-vinyl)-2,2-dimethylcyclopropane-carboxylic; 2-ClBA: acid 2-(4-chlorophenyl)-3-methylbutyric; Cl_2_CA : acid 3-(2,2dichlorovinyl)-2,2-dimethylcyclopropane-carboxylic; ClCF_3_CA: acid 3-(2-chloro-3,3,3-trifluoro-1-propenyl)-2,2-dimethylcyclopropane-carboxylic; 2,4-D: acid 2,4-dichlorophenoxyacetic; 2,4-DB: acid 2,4-dichlorophenoxybutanoic; 4F3PBA: acid 4-fluoro-3-phenoxybenzoic; MCPA: acid 2-methyl-4-chlorophenoxyacetic; MCPB: acid 2-methyl-4-chlorophenoxybutanoic; 3Me4NP: 3-methyl-4-nitrophenol; 3-PBA: acid 3-phenoxybenzoic; TCPy: 3,5,6-trichloro-2-pyridinol.

As a whole, 51 BRPs over 67 analyzed (76%) were detected in small mammal hair, with 27 parent chemicals detected out of 39 screened (67%) and 25 metabolites detected out of 28 (89%) (Table 1). Thirteen compounds were present in more than 75% of individuals: DMP, PNP, 1-(3,4-dichlorophenyl)urea, DEP, PCP, 3Me4NP, 1-(3,4-dichlorophenyl)-3-methylurea, DETP, fipronil, fipronil sulfone, trifluralin, DMTP and HCB. Most of them are transformation products of organochlorine, organophosphorous, urea and phenylpyrazole pesticides. Then, the proportion of detection rapidly dropped under 25% of the samples. Only three compounds were detected in 50–75% of the individuals (Table 1: lindane γ-HCH (organochlorine insecticide), terbutryn (triazine/triazinone herbicide) and fenuron (urea herbicide). Five substances were found in 25–50% of the animals: DMST (metabolite of tolylfluanide, an amide fungicide), flusilazole (azole fungicide), α-endosulfan (organochlorine insecticide), DMDTP (organophosphorous insecticide metabolite) and diuron (urea herbicide). The 10 highest measured concentrations ranged between 30 and 118 ng/g, and were mostly represented by DMP (seven of the 10 values) together with PNP and 1-(3,4-dichlorophenyl)urea. Seven compounds exhibited concentrations higher than 10 ng/g, which were the same as the most frequent: DMP, PNP, 1-(3,4-dichlorophenyl)urea, DEP, PCP, 3Me4NP, plus DEDTP (organophosphorous metabolite, 6% of individuals). Considering the 16 BRPs that have never been detected, 13 were parent pesticides and three were metabolites, distributed in one fungicide, three herbicides, and 12 insecticides/biocides. The non-detected compounds belong to several chemical families including organochlorines, organophosphorous, carbamate, and urea pesticides.

A total of 61 CUPs out of 73 analyzed were detected in small mammal hair, with 54 parent pesticides out of 66 tested (82%) and seven metabolites detected out of seven screened (100%) (Table 2). Many of the detected CUPs were found in a large proportion of individuals: 25 compounds were detected in more than 75% of the individuals, which means that 41% of the 61 detected CUPs were present in 75–100% of individuals. These 25 most frequently detected compounds belonged to various chemical families and all uses of CUPs (Table 2). The herbicides belonged to the families of organochlorines (metolachlor and metazachlor), acid herbicides (MCPA, 2,4-d,dichlorprop and mecoprop), thiocarbamates (prosulfocarb), amide pesticides (dimethachlor), uracils (lenacil), and dinitroaniline (pendimethalin). The fungicides were of the main families strobilurines (azoxystrobin and pyraclostrobin), azoles (tebuconazole, epoxiconazole, thiabendazole, prochloraz, and propiconazole; cyproconazole in 73% of individuals), carbamates (carbendazim) and carboxamides (boscalid). The most frequently detected insecticides were mainly metabolites of pyrethroids (3-PBA, Cl_2_CA, and ClCF_3_CA), as well as neonicotinoids (thiacloprid and imidacloprid) and the specific metabolite of chlorpyrifos TCPy (3,5,6-trichloro-2-pyridinol; organophosphorous pesticide). Noticeably, the five herbicides isoproturon (urea), propyzamide (benzamide), chlortoluron (urea), oxadiazon (oxadiazin) and diflufenican (carboxamide), as well as the fungicide trifloxystrobin (strobilurin) and the insecticide cypermethrine (pyrethroid), were detected in at least 50% of the samples (Table 2). Five more compounds were detected in 25–50% of animals: zoxamide (benzamide), difenoconazole (azole), cyhalothrin and Br_2_CA (pyrethroids), and 2,4-DB (acid herbicide). The 10 highest measured concentrations ranged from 200 to 500 ng/g, which were far higher than for BRPs. These high concentrations were found for the fungicides boscalid, carbendazim, and prochloraz and the herbicides dichlorprop, MCPA, and propyzamide. A greater number of compounds exhibited higher concentrations than observed for BRPs, since 29 compounds presented concentrations higher than 10 ng/g. Moreover, 16 compounds were quantified at higher levels than 50 ng/g, and 10 compounds at higher levels than 100 ng/g (Table 2). The 10 compounds that had the highest concentrations were the herbicides propyzamide, MCPA, dichlorprop, diflufenican, mecoprop, and metolachlor, and the fungicides boscalid, epoxiconazole, carbendazim, and prochloraz. They were not all among the most detected compounds (Table 2). Six compounds exhibited concentrations ranging from 50 to 100 ng/g: the insecticide imidacloprid, the herbicides aclonifen and isoproturon, and the fungicides cyproconazole, propiconazole and tebuconazole. Various chemical families are represented among the CUPs exhibiting high concentrations in small mammals, including carbamates, carboxamids and benzamids, acid and urea herbicids, azoles and neonicotinoids (Table 2). The insecticides showed concentrations overall lower than herbicides and fungicides, since no value above 50 ng/g was measured within insecticides except for imidacloprid. Besides the neonicotinoid imidacloprid, the insecticides showing the highest values (> 10 ppb) were all pyrethroids, either parents or their metabolites (cyfluthrine, cyhalothrine, permethrine, 3-PBA, Br_2_CA, Cl_2_CA). Among the 12 CUPs that have never been detected, only parent compounds were present, with six fungicides, two herbicides and four insecticides belonging to various chemical families such as azole, carbamate, organophosphorous, triazine, neonicotinoid, strobilurine, oxadiazine and urea pesticides.

A significant positive relationship was found between detections of CUPs in small mammal hair samples and the quantities of pesticides sold in 2016 in the Region were the ZAPVS is located (i.e. Deux-Sèvre, where most of small mammals in this study were captured and analyzed) (Spearman's rho = 0.66, *p-value* < 0.001, Supplementary Fig. S1). The number of quantification above 10 ng/g in hair of small mammals was also found significantly positively associated to the quantities of each corresponding pesticide sold in 2016 in Deux-Sèvres (Spearman's rho = 0.58, *p-value *< 0.001, Supplementary Fig. S1).

### Number of compounds and concentrations according to species, habitat, zone and farming

**Figure 1:**
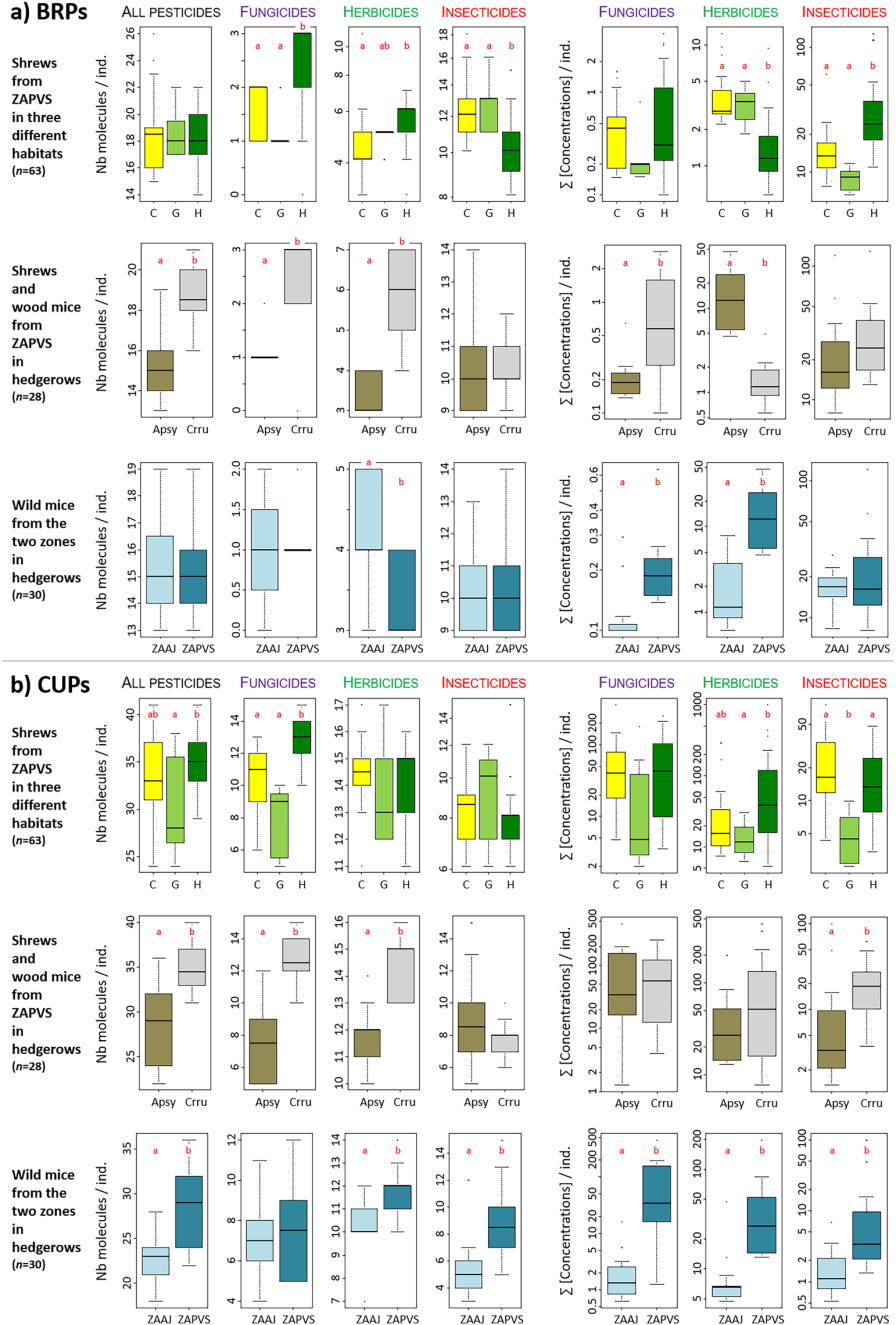
Boxplots of number of molecules and sum of concentrations for (**a**) banned and restricted pesticides (BRPs) and (**b**) currently used pesticides (CUPs) according to habitats, species and sites. Statistical differences between groups are indicated by lower case letters, different letters indicate statistically significant differences between factor levels (statistical significance: p-value< 0.05). *C* cereals, *G* grasslands, *H* hedgerows, *Crru* *Crocidura russula* shrew, *Apsy* *Apodemus sylvaticus* wood mouse, *ZAAJ* Zone Atelier Arc Jurassien, *ZAPVS* Zone Atelier Plaine et Val de Sèvre.

Overall, between 32 and 65 compounds were detected in each individual (mean ± SD = 49 ± 7, median = 50). Considering shrews and mice separately, the average (± SD) reached 52 ± 5 compounds in shrews (min–max = 41–65), and 41 ± 5 compounds in mice (min–max = 32–52). Specifically, 13–26 BRPs (median = 17; mean ± SD = 17 ± 3) and 18–41 CUPs (median = 32; mean ± SD = 31 ± 6) were detected in each hair samples (Fig. 1, Supplementary Tables S1–S2). Considering BRPs only, small mammal individuals exhibited mixtures constituted of at least three herbicides and eight insecticides (mean ± SD = 2 ± 1 fungicides, 5 ± 1 herbicides, and 11 ± 2 insecticides). Considering CUPs only, animals exhibited mixtures constituted of at least four fungicides, seven herbicides and three insecticides (mean ± SD = 10 ± 3 fungicides, 13 ± 2 herbicides, and 8 ± 2 insecticides).

Overall, shrews showed a higher contamination than mice considering both the number of compounds, and to a lower extent, the concentrations (Fig. 1, Supplementary Tables S1–S2). Shrews exhibited higher number of compounds for total of all BRPs and all CUPs, for fungicides, and for herbicides. However, similar number of insecticides were measured in shrews as in wood mice (Fig. 1, Supplementary Tables S1–S2). Shrews showed greater concentrations of pesticides, though significantly only for banned fungicides and current insecticides, with the exception of banned herbicides for which mice had higher concentrations (Fig. 1, Supplementary Tables S1–S2).

Shrews captured in hedgerows and cereal crops generally exhibited the highest number of compounds or sum of concentrations for both BRPs and CUPs, while the lowest in grasslands. Exceptions were noticed for the number of banned insecticides and the concentrations of banned herbicides, for which animals from hedgerows showed the lowest levels (Fig. 1, Supplementary Tables S1–S2). However, such a pattern of higher concentrations or number of compounds in hedgerows and/or cereals was not always significant (Fig. 1, Supplementary Tables S1–S2). The number of compounds was significantly higher in individuals captured in hedgerows than in cereals and/or in grasslands for banned fungicides and herbicides, all CUPs, and current fungicides. Concentrations were significantly greater in animals captured in hedgerows than in cereals and/or in grasslands for current herbicides and insecticides, as well as banned insecticides. The number of compounds and concentrations were never significantly higher in grasslands than in cereals. They were significantly higher in animals from these two habitats than in hedgerows only for the number of banned insecticides and the concentrations of banned herbicides (Supplementary Tables S1–S2). The variability between individuals was the greatest in hedgerows, with animals showing number of compounds and concentrations both among the lowest and the highest (Fig. 1).

Few differences were found in mice contamination by BRPs between the two sites of sampling ZAAJ and ZAPVS. Mice from ZAAJ showed a slightly higher number of banned herbicides, but significantly lower concentrations of banned fungicides and herbicides than mice from ZAPVS. The pattern was clearly different for CUPs, for which mice from ZAPVS exhibited higher number of compounds and greater concentrations than mice from ZAAJ, but the difference was not significant for current fungicides (Fig. 1, Supplementary Tables S1–S2).

**Figure 2:**
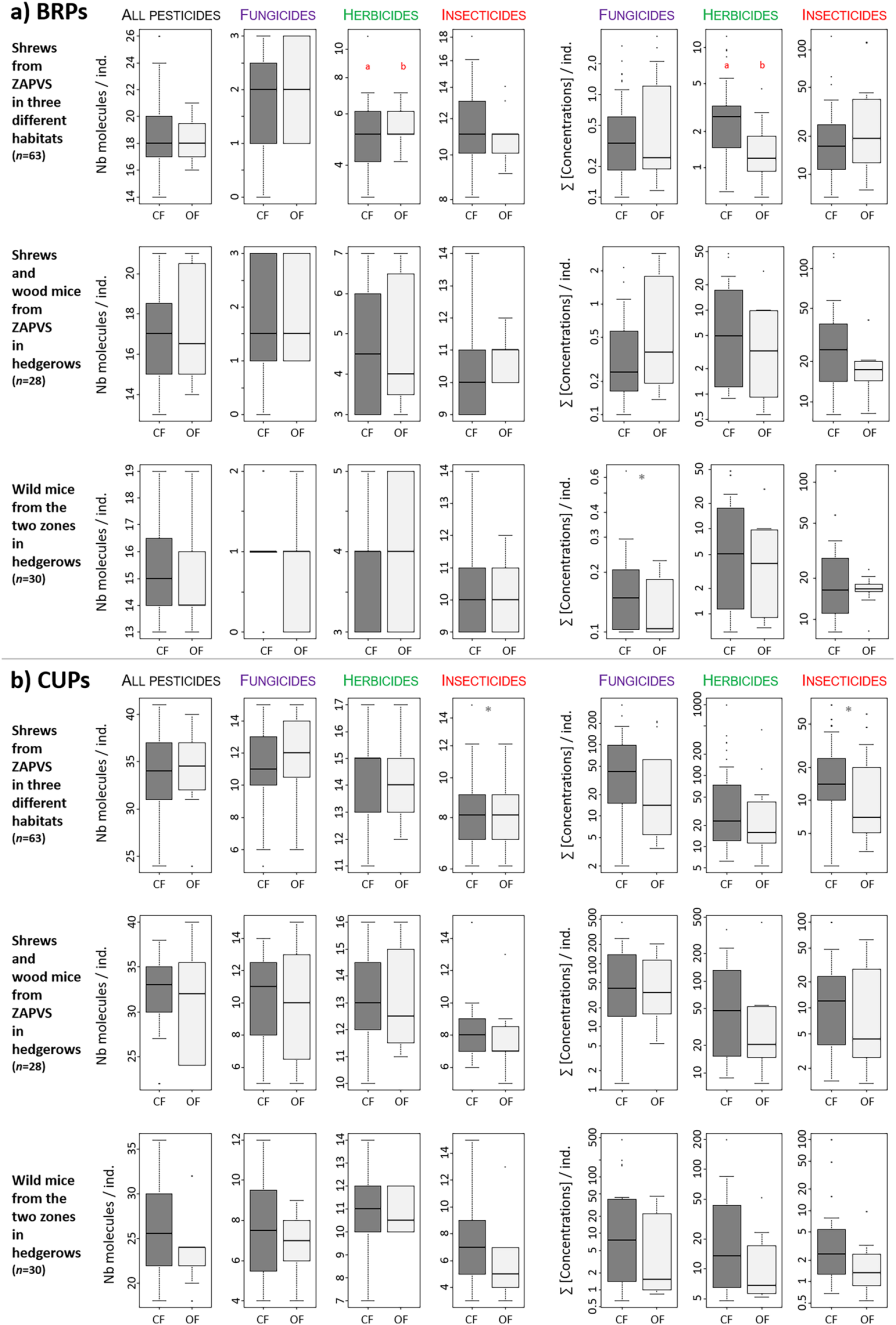
Boxplots of number of molecules and sum of concentrations for (**a**) banned and restricted pesticides (BRPs) and (**b**) currently used pesticides (CUPs) according to farming practices. Statistical differences between groups are indicated by red asterisks (statistical significance: p-value< 0.05), if close to significance level (0.10 > p-value > 0.05) the color of asterisk is grey. *CF* Conventional farming, *OF* Organic farming.

Number of BRPs and their concentrations were overall similar between organic and conventional farming, except for herbicides in shrews from ZAPVS, which showed higher number and concentrations in conventional than in organic farming contexts (Fig. 2, Supplementary Tables S1–S2). However, this result might be considered cautiously since metabolites of urea herbicides were included in the class of BRPs because most of these herbicides are outlawed, but a few urea compounds were still authorized in 2016 (Tables 2 and 3). Recent use might thus confound this result on BRPs. While a general trend of larger number and concentrations of CUPs in conventional than in organic contexts might be noticed numerically and graphically (Fig. 2, Supplementary Tables S1–S2), we did not detect any significant differences between CF and OF for the CUPs analyzed in this study.

**Table 3:** Overview of small mammal sample size.

Site	Habitat	Type of farming	Wild mice		Shrew	Total
			*Apodemus flavicollis*	*Apodemus sylvaticus*	*Crocidura russula*	
ZAAJ	Hedgerows	Conventional	5	5		10
		Organic	3	3		6
	Total		8	8		16
ZAPVS	Cereals	Conventional			21	21
		Organic			1	1
		Total			22	22
	Grasslands	Conventional			4	4
		Organic			3	3
		Total			7	7
	Hedgerows	Conventional		10	22	32
		Organic		4	12	16
		Total		14	34	48
	Total			14	63	77
Total			8	22	63	93

### Contamination profile according to habitats, species and sites

**Figure 3:**
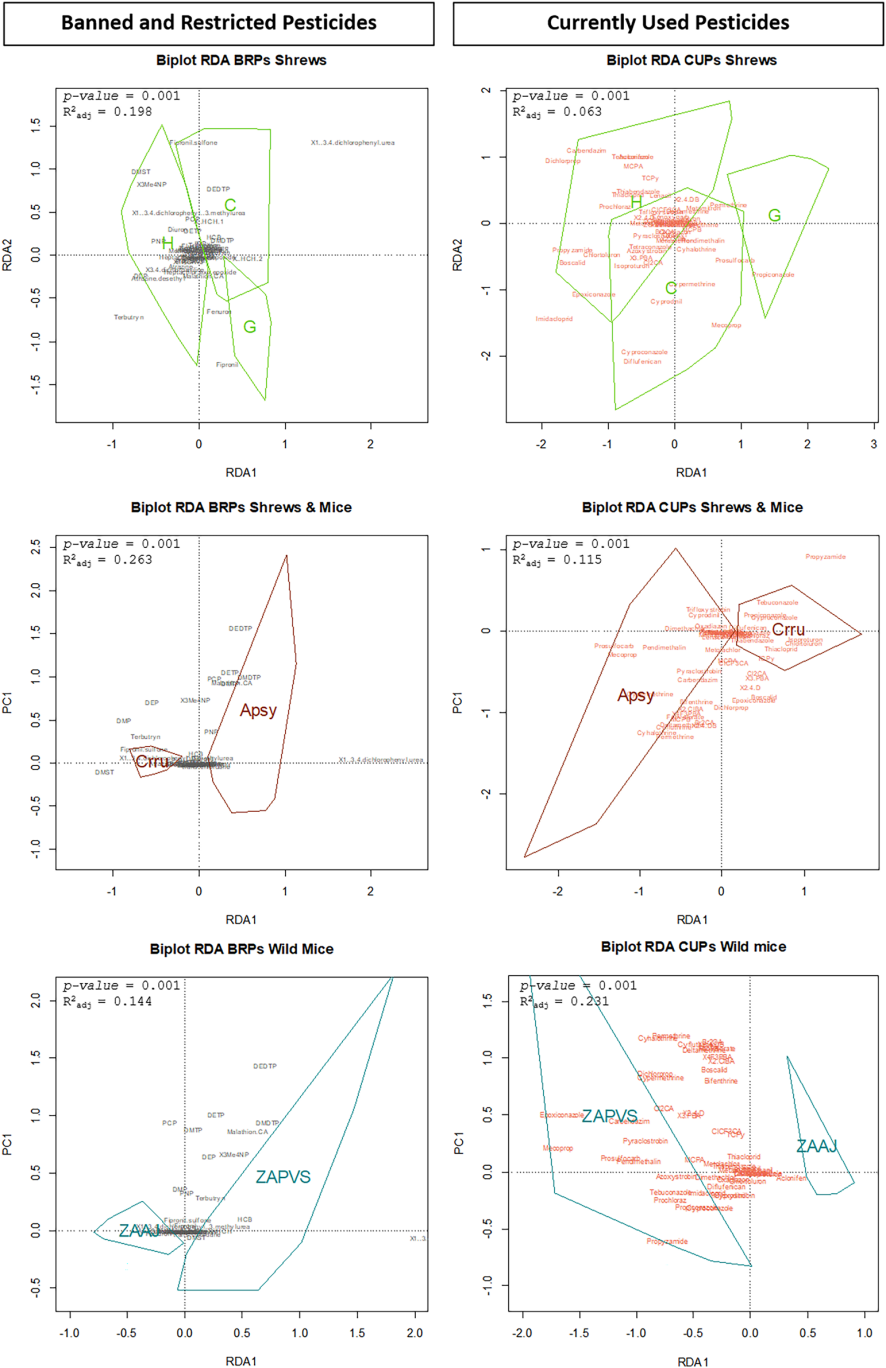
Influence of habitat, species, and sampling site on small mammal contamination by pesticides: correlation biplots of redundancy analyses. The factor “organic farming” is not displayed since not statistically significant (*p-value* > 0.05). *C* = cereals, *G* = grasslands, *H* = hedgerows; *Crru* = *Crocidura *russula shrew, *Apsy* = *Apodemus sylvaticus* wood mouse; *ZAAJ* = Zone Atelier Arc Jurassien; *ZAPVS* = Zone Atelier Plaine et Val de Sèvre; R^2^_adj_: adjusted R squared. Groups are dispayed according to convex hulls. See Tables 1 and 2 for detailed meaning of compound acronyms.

Multivariate analyses showed that profiles of both BRPs and CUPs differed significantly between habitats, species and zones (Fig. 3). The profiles of compounds were slightly to moderately explained by the factors tested considering the determination coefficients, which ranged from 0.063 to 0.263. Organic farming was never retained as an explanatory factor in models (*p-value* > 0.05 and delta AIC > 2), meaning that profiles did not differ significantlty between animals captured in OF or CF contexts.

Profiles of pesticide residues were significantly correlated to the type of habitat (*p-value* = 0.001), with 19.8% and 6.3% of variance explained for BRPs and CUPs, respectively. Profiles of BRPs in hedgerows were dominated by relatively high concentrations of DMST, 3Me4NP, 1-(3,4-dichlorophenyl)-3-methylurea, PCP, diuron, DETP, PNP, atrazine, 3,4-dichloroaniline, DEP, and atrazine desethyl (Fig. 3). In cereals, profiles were associated to high levels of fipronil sulfone, DEDTP, lindane, HCB, and DMDTP while grasslands showed profiles associated with fenuron and fipronil. For CUPs, contamination in cereals was characterized by high levels of cyproconazole, diflufenican, cyprodinil, mecoprop, cypermethrin, prosulfocarb, cyhalothrin, pendimethalin, and permethrin. Profiles of CUPs in hedgerows were associated with high levels of carbendazim, dichlorprop, aclonifen, tebuconazole, MCPA, TCPy, thiabendazole, thiacloprid, lenacil, prochloraz, propyzamide, chlortoluron, boscalid, and epoxiconazole. In grasslands profiles were characterized by the concentration of propiconazole.

Profiles of residues significantly differed according to the species studied (*p-value* = 0.001), which explained 26.3% and 11.5% of the variance for BRPs and CUPs, respectively. Shrews differed from mice with higher levels of DMST, terbutryn, 1-(3,4-dichlorophenyl)-3-methylurea and fipronil sulfone while mice exhibited profiles associated with higher levels of DEDTP, DETP, DMTP, DMDTP, PCP and malathion ca (Fig. 3). High levels of tebuconazole, propiconazole, cyproconazole, diflufenican, prochloraz, thiabendazole, isoproturon, chlortoluron, thiacloprid, and TCPy were characteristics of profiles in shrews while mice profile was associated to trifloxystrobin, cyprodinil, oxadiazon, dimethachlor, lenacil, pendimethalin, prosulfocarb, mecoprop, pyraclostrobin, carbendazim, MCPB, cypermethrin, bifenthrin, cyhalothrin, cyfluthrin, fenvalarate, deltamethrin, and metabolites of pyrethroids.

The contamination significantly differed according to the sampling zone (*p-value* = 0.001), which explained 14.4% and 23.1% of variance for BRPs and CUPs, respectively. Contamination in mice from ZAPVS was associated with high levels of DMDTP, malathion carboxylic acid, 3Me4NP, HCB, and 1-(3,4-dichlorophenyl)urea. Mice from ZAPVS showed, overall, higher concentrations of CUPs than in ZAAJ. The contamination profiles in ZAPVS was dominated by high levels of epoxiconazole, carbendazim, pyraclostrobin, mecoprop, prosulfocarb, pendimethalin, azoxystrobin, tebuconazole, imidacloprid, prochloraz, propiconazole, and propyzamide (Fig. 3).

### Hierarchy of determinant factors on contamination profiles and discriminating pesticides

**Figure 4:**
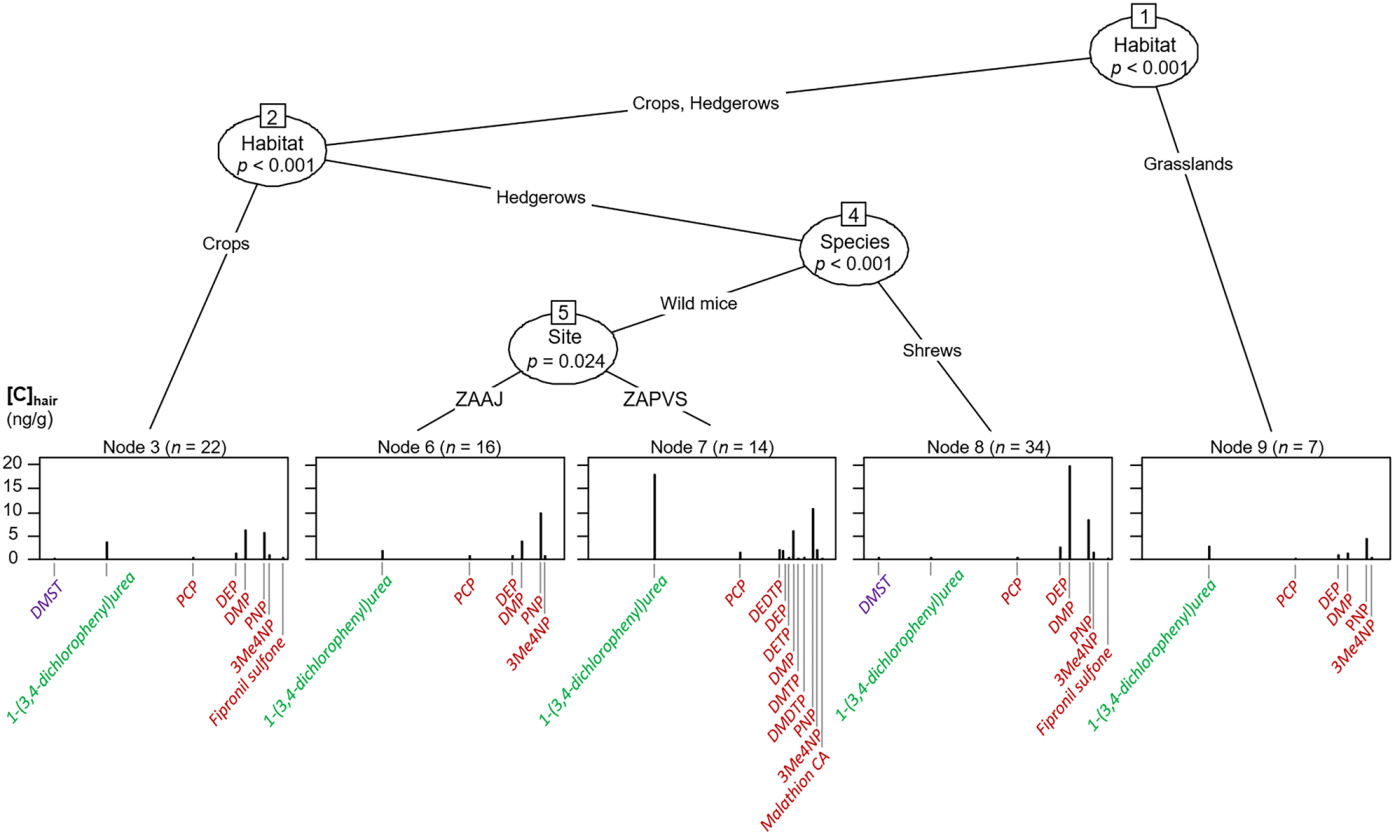
Multivariate conditional inference tree showing the factors significantly splitting the profiles of contamination by banned and restricted pesticides. *ZAAJ* = Zone Atelier Arc Jurassien; *ZAPVS* = Zone Atelier Plaine et Val de Sèvre. The names of fungicides are written in purple, those of herbicides in green and those of insecticides in dark red. The names of metabolites are in italics. See Table 1 for detailed meaning of compound acronyms.

**Figure 5:**
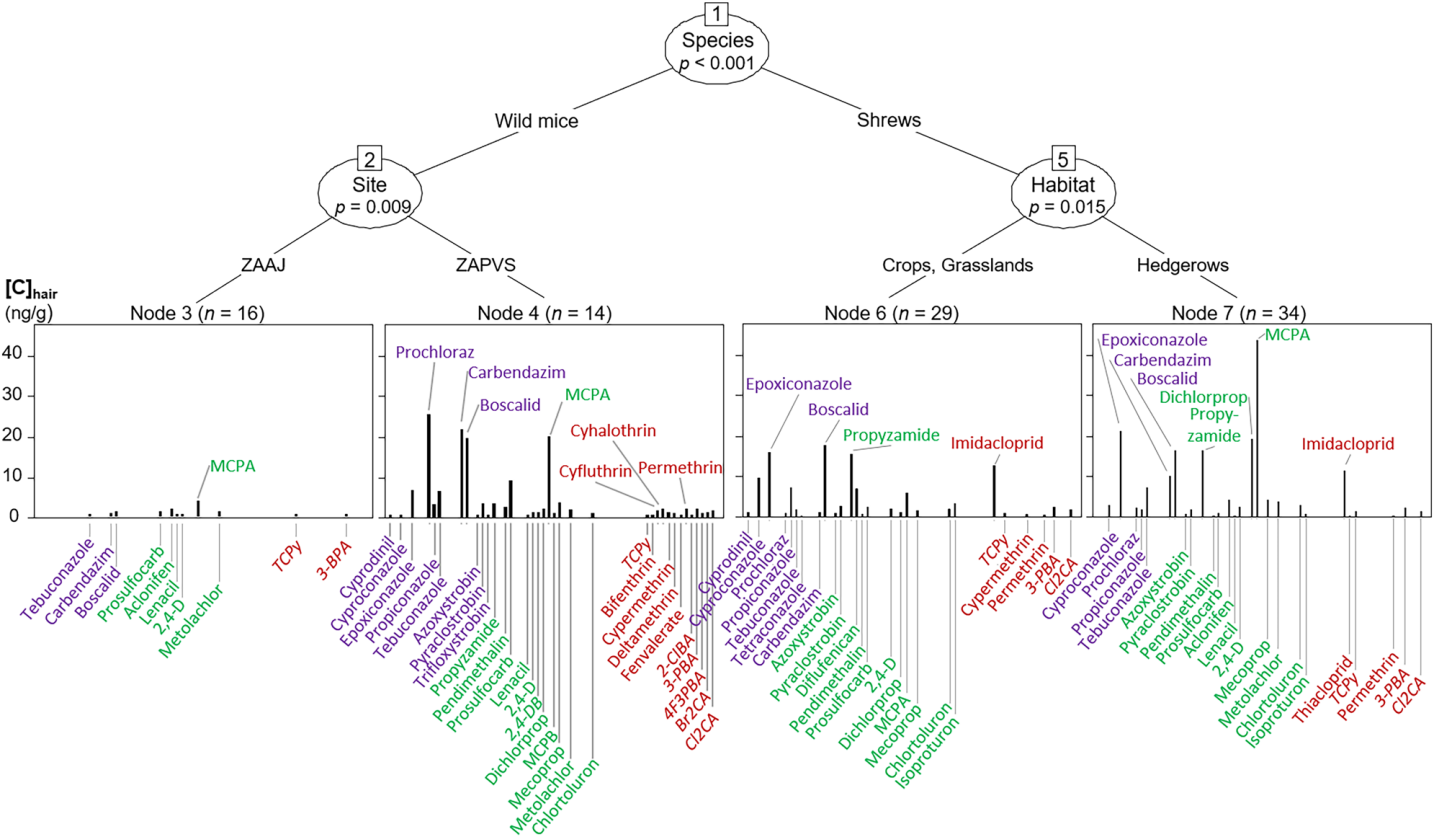
Multivariate conditional inference tree showing the factors signifcantly splitting the profiles of contamination by currently used pesticides. *ZAAJ* = Zone Atelier Arc Jurassien; ZAPVS = Zone Atelier Plaine et Val de Sèvre. The names of fungicides are written in purple, those of herbicides in green and those of insecticides in dark red. The names of metabolites are in italics. See Table 2 for detailed meaning of compound acronyms.

Inference trees allowed displaying the compounds that characterized the contamination profiles of small mammals, showing the mixtures of pesticides that small mammals accumulated and thus were exposed to. Conditional inference trees showed that the factor with the strongest association with contamination profiles differed between BRPs and CUPs: while profiles firstly differed according to habitat for BRPs, they were split according to species for CUPs (Figs. 4 and 5). The type of farming “organic” or “conventional” did not provide any significant splitting of the multivariate dataset.

For BRPs, profiles in grasslands were characterized by both low number of compounds and low concentrations (Fig. 4). Further, profiles of pesticides were discriminated for crops where a higher number of molecules and higher concentrations were found such as for the fungicide DMST, the metabolite of urea herbicides 1-(3,4-dichlorophenyl)urea and several insecticides (e.g. PCP, DEP, DMP, PNP). The profiles in hedgerows were segregated between shrews and mice, with greater number of insecticides in mice but higher concentrations of insecticides in shrews. Moreover, greater concentrations of urea herbicide were found in mice than in shrews, and a high load of the fungicide DMST were observed in shrews only. Finally, profiles in mice from ZAAJ were characterized by less compounds than in mice from ZAPVS and generally lower concentrations (Fig. 4).

Profiles of CUPs were split between shrews and mice, but showed a common pattern of high number of molecules in the two groups of species, and elevated concentrations for several fungicides, herbicides and insecticides (Fig. 5). In shrews, concentrations reached overall higher levels, especially for insecticides and notably imidacloprid (Fig. 5). Many CUPs were common to both species, although the compounds presenting the highest concentrations were different. As an example, in mice, many pyrethroids and their metabolites were quantified, while in shrews less compounds showed high concentrations but the concentrations reached were greater. The profiles of CUPs in mice were split between the two sampling zones, the contamination being discriminated by a lower number of molecules and overall lower concentrations in ZAAJ than ZAPVS. In shrews, the profiles of CUPs were separated according to the habitats, with open habitats crops and grasslands opposed to hedgerows. In both cases, shrews exhibited high concentrations for a large number of compounds, and this was observed in the different habitats in the case of epoxiconazole, boscalid, propyzamide, 2,4-d, imidacloprid and 3-BPA for instance. Moreover, profiles in shrews from hedgerows showed high concentrations of some other compounds such as carbendazim, dichlorprop, metolachlor, and MCPA, as well as several pesticides at lower levels not evidenced in profiles from other habitats like thiacloprid, aclonifen and lenacil. Some CUPs were mostly found in shrews captured in crops or grasslands such as cyprodinil, tetraconazole, diflufenican, and cypermethrin (Fig. 5).

## Discussion

As expected due to the pervasiveness of environmental contamination of BRPs, a general contamination was characterized in small mammal hair, with 76% of the screened compounds detected at least once and 15 compounds detected in half or more of the individuals. These results show that, although emissions and environmental concentrations in air or water showed overall decreasing trends since the ban of the pesticides^51^, numerous BRPs are still present in agricultural areas and food webs at detectable and quantifiable concentrations. Banned pesticides that persist in soils might be remobilized due to current practices and global change, as shown for instance for DDT stored in vineyard soils, subjected to release because of the use of postemergence herbicides like glyphosate that induced an increase in soil erosion^9^. The most frequently detected compounds, or exhibiting the highest concentrations, were both parent pesticides and metabolites. They belonged to various chemical families, were used as insecticides, herbicides or fungicides, and showed dates of ban ranging from the 80’s to the most recent in the 2000s. It is therefore impossible to picture general patterns about usage or chemical drivers of legacy compounds transfer and impacts in ecosystems nowadays. Illegal use could be one of the causes of the contamination of small mammals by BRPs. Cases have been reported in ZAPVS for fipronil and lindane (V. Bretagnolle, personal communication). In the 2000s, the illegal use of anticholinesterase organophosphorous and carbamate insecticides has been reported as a frequent cause of wildlife poisoning in Europe^25^. Such illegal practices are still occurring nowadays, leading to the exposure of both birds and mammals to banned pesticides^52–54^ and even endangering wildlife populations in Europe and worldwide^55–57^.

A few compounds are now banned or submitted to new regulation but were still used during the 2010s: carbendazim, dimethoate, neonicotinoids, isoproturon and linuron for instance. Our data therefore represent a baseline of wildlife exposure before the ban, to survey the fate of environmental contamination and exposure of fauna in the coming decades.

Contrary to our expectations, the concentrations of CUPs in small mammals did not exhibit a pattern of “hot-spots” of exposure in specific cases like treated plots or particular individuals. Instead, a pervasive exposure to dozens of substances was evidenced in all small mammal species and relatively high concentrations were quantified in many individuals. One would hypothesize that the magnitude of exposure of small mammals to specific CUPs may be related to their magnitude of use and timing of application both at field level and within the agricultural ecosystem. Since contamination was assessed via measurements of pesticide residues in hair, comparisons of the concentrations between different compounds must be interpreted cautiously. In the same way as for any other biological matrix, depending on compound specific bioavailability, equivalent external exposure to different compounds may lead to different internal dose^45^. Even if the concentrations in hair are known as proportional to the level of exposure, due to differences in physico-chemical properties of the pesticides and potential differences in routes of exposure, the toxicokinetics of the various compounds can be markedly different between each other. As a result, the concentration of chemicals in the environment cannot be directly extrapolated from their concentration in animals’ hair, but their presence in the animals remains a proof of exposure. Considering contamination profiles rather than specific compounds, our results suggested the role of CUP application intensity and timing in the exposure of small mammals. Indeed, differences were found between mice captured around maize fields and grasslands in ZAAJ and mice captured around winter cereals in ZAPVS. Comparing the official sales of pesticides in 2016^58^ in the region Deux-Sèvres (where small mammals were mostly captured in our study), and using quantities sold as a proxy of magnitude of usage since no more detailed information was available, the intensity of CUP use seemed to play a significant role in shaping general exposure of small mammals. Both detection and quantification of CUPs in small mammals were significantly positively related to the quantities sold (Supplementary Fig. S2), and the ranking of detection frequency matched the ranking of sold quantities (Supplementary Table S3). Several pesticides rarely or never detected in our study (e.g. oxamyl, penconazole, imazalil, dinotefuran, dimethoate) were accordingly not listed in the sales in 2016 (n.b. penconazole and dimethoate were listed in 2015). Overall, the CUPs within the 30% most detected were also molecules within the 30% most sold in quantities, but with noticeable exceptions. For instance, lenacil, thiabendazole and carbendazim were among the CUPs frequently quantified here, but they were sold in relatively small quantities in the region or even not sold. Interestingly, the conclusions of Bro et al. (2016) raised comparable insights. Several compounds were detected in partridge clutches whereas not associated to potential exposure of the female, but also an absence of detection of compounds in eggs when exposure was not expected was noticed. Matches between expected exposure and detection were found for cyproconazole, cyhalothrin, prochloraz, fenpropidin, and tebuconazole. Moreover, several compounds were detected while known to have been used over the study site such as bromoxynil, thiametoxam (+ clothianidin), and diflufenican. Such a list of CUPs for which treatments concured with accumulation in wildlife overlap the findings of the present study.

For CUPs, lower persistence in the environment is expected than for legacy pesticides, which half-lives are longer. However, accumulation in the environment and in biota may not only depend on the properties of the compounds and of the media but also on the intensity and recurrence of use. Recently the concept of “pseudo-persistence” has been proposed, where “*repeated use of [pesticides] can lead to their gradual accumulation in the environment (“pseudo-persistence”) as their degradation is slower than their input to the environment*”^10^. Such a process of pseudo-persistence may partly explain the ubiquitous and general accumulation of CUPs in the present study.

With regards to the ecological traits of small mammals, the granivorous wild mice and the insectivorous/vermivorous shrews show differences in contamination profiles. However, the expected pattern of higher concentrations of BRPs in shrews, which are known to biomagnify for many compounds analyzed here^59^, is not straightforward. Besides, currently used insecticides that were not supposed to biomagnify showed higher concentrations in shrews than in mice. Further, the different patterns of contamination between shrews and mice were not segregated according to type of use (i.e. fungicide, herbicide or insecticide). Such a mismatch with our hypothesis might be due to the fact that *Apodemus* mice are not strictly herbivorous and granivorous rodents, a significant part of their diet being composed of animal matter^60^. Transfer in food webs of both BRPs and CUPs may be one of the processes involved in accumulation of the compounds in both rodents and shrews. A foremost concern is that such a trend for omnivory is a widespread trait in wildlife^61^. Besides, the differences in contamination profiles may result from behavioural difference (e.g. time spent in fields, mobility and burrowing), physiological differences (e.g. metabolism) and variations in toxicokinetics between the rodent and shrew species.

Both taxa exhibit contamination by pesticides that can be applied via spraying, seed coating and/or granules spreading, which questions the pathways of exposure that can be oral via direct consumption and watering or grooming, trophic transfer, inhalation, and/or dermal contact. The oral route is considered as the major one in wildlife^62^, and ingestion of contaminated food is the pathway considered in risk assessment process for birds and mammals^63^. Transfer of pesticides in food webs has been evidenced decades ago in the case of “old” pesticides such as organochlorines^1^, but has far less been investigated for CUPs. Granivores can be directly exposed to CUPs via the ingestion of treated seeds, as shown for many farmland birds as well as mammals like hare and wood mouse^17,23,50,64^. Moreover, herbivores may be exposed through consumption of vegetation organs, since cultivated and wild plants have been shown to be contaminated by systemic insecticides^49^. Both BRPs and CUPs have been found to accumulate in earthworms, and in insects constituting the boluses of bird nestlings^11,65–67^. Pesticide overspray can also be an exposure pathway for wildlife, with potential uptake via dermal and respiratory routes but also oral route due to grooming^68,69^. Our results thus raised important questions about the relative contribution of exposure sources and pathways, an area of research that is still difficult and understudied in wildlife but strongly needed to improve exposure and risk assessment.

Direct comparisons with publications dealing with the monitoring of pesticides in wildlife in terms of number of compounds or frequency of detection and concentrations were hampered by differences in the list of screened substances, in analytical method performances, and/or in the type of matrix analyzed. Moreover, only few studies addressed multi-residue screening of CUPs or of both legacy and current pesticides. Only general pictures can be highlighted. Overall, the legacy pesticides detected here and in wildlife such as birds^70,71^, bats^19^, game mammals^72^, and amphibians^18,73^ in Europe and America were mostly organochlorines, organophosphates, carbamates and triazines/triazinones. These compounds together with anticoagulant rodenticides are frequently included in monitoring schemes since they are known to be transferred in food webs and often reported as causes of intoxications^25,43^.

By providing the first data on wild rodents and shrews, our results provide additional evidence of the exposure to and accumulation of neonicotinoids in many trophic groups worldwide, as shown in birds including nectarivores^74^, granivores and omnivores^50,75^, insectivores and birds of prey^66,76^. Some recent studies evidenced, as here, the detection of various CUPs in wildlife. Current pesticides such as azole, piperidin and strobilurin fungicides, as well as chloroacetanilid, dinitroaniline, benzonitrile, carboxamid and acide herbicides, and also pyrethroid and neonicotinoid insecticides have been indeed found in our work and also in eggs or liver of birds^70,71^, tissues of bats^19^, of wild boar, roe deer and red deer^72^, and of amphibians^18,73^. Considering studies which addressed large multi-class residue screening as done here, the number of compounds detected reached the same order of magnitude than in our study with 52 contaminants found in a screening conducted on 15 different bird species from the Canary Islands^71^, with 87 compounds detected out of 322 targeted in a screening realized on two bat species in Turkey showing on average 26 compounds by individual^19^, and with 28 compounds detected over 460 screened in wild boars, roe deers and red deers from northeastern Poland^72^.

In our results, shrews exhibited overall a higher number of compounds than wood mice. In a study where the concentrations of 30 CUPs were analyzed in carabid beetles sampled in the ZAPVS in 2016^77^, individuals of the generalist predator *Poecilus cupreus* exhibited a greater number of compounds than phytophagous beetles of the species *Harpalus dimidiatus*. In a recent study dealing with analysis of POPs, currently used and banned pesticides, rodenticides, and pharmaceuticals in 15 bird species, the species exhibiting the largest number of compounds detected were predators like the long-eared owl *Asio otus* and the common kestrel *Falco tinnunculus*^71^. The birds having the lowest number of compounds detected were mostly omnivorous species such as the European blackbird *Turdus merula* species or the common raven *Corvus corax*^71^. Altogether, our findings and those reported above suggest an increase of the number of compounds wildlife is exposed to and accumulated at higher trophic levels in food webs and we propose a new concept referred to as “biowidening”, to describe such phenomena. Biomagnification depicts the process by which contaminant concentrations increase in tissues of organisms along food webs, with a trophic enrichment of the compound and progressive rise of concentrations with increment of trophic level. Biowidening would describe a trophic enrichment of the panel of compounds and progressive increase in diversity of compounds organisms are exposed to with the increment of trophic level. Further studies are encouraged to investigate and validate, if relevant, such a potentially new notion.

The use of multi-residue analyses reveals the accumulation of complex mixtures of compounds in wildlife, which reflects a co-exposure that might be caused by simultaneous, successive or cumulative multiple exposure^78^. The ecotoxicological significance of such a co-exposure might be due to either chronic long-term exposure to low doses, or acute short-term exposure to peaks but possibly recurrent, or both. The CUPs quantified in our work can have toxic effects, as known from the literature and investigated in marketing authorization. However, it is hard to interpret hair concentrations with regards to toxicity benchmarks because (1) relationships between residues in hairs of wild small mammals and exposure doses are not fully characterized, (2) time sequence in exposure are unknown, and (3) toxicity thresholds have been established usually under single compound exposure^79^, an assumption that is not fulfilled here. The present results provide valuable information from a global environmental contamination perspective since they allow identifying, at least partly, the mixtures of compounds that may be of concern for transfer in food webs and ecotoxicological impacts in wildlife.

Altogether, our results and recent findings showed that exposure to complex mixtures of BRPs and CUPs is likely to be the rule rather than the exception. First of all, these reports raise issues about the toxicity of mixtures of pesticides on wildlife populations, and subsequent impacts on communities and biodiversity. Besides, the global contamination of granivorous, omnivorous and insectivorous species questions the potential of CUP transfer in food webs, exposure of predators and top-predators and potential for biomagnification and/or biowidening of some compounds. Focusing on CUPs, direct intoxication through consumption of treated seeds has mostly been studied in granivorous/omnivorous birds^17^, while trophic cascade effects (via reduction of resources) have predominantly been investigated in populations of insectivorous birds and bats (e.g.^80,81^), and secondary poisoning of predators has mostly been evidenced for specific pesticides such as anticoagulant rodenticides^37^. Cascading effects of CUP in food webs, through the transfer of compounds in biota and via direct toxic effects and/or indirect effects on communities and trophic interactions are therefore raised. The potential role of small mammals as essential preys in agro-ecosystems both in the propagation of pesticide impacts through the reduction of their populations and through predator exposure to mixtures of pesticides requires further investigation.

Ubiquity of exposure to CUPs in both treated and untreated habitats was evidenced for soils, earthworms and beetles over the ZAPVS^11,77^. However, soils and earthworms showed an overall lower CUP accumulation in grasslands and hedgerows than in arable crops, and a lower CUP accumulation in plots under OF than under conventional farming^11^. An ubiquitous, general, exposure of house sparrows (*Passer domesticus*) to neonicotinoids in Swiss farmlands has been shown, but birds living in conventional farms exhibited higher concentrations than individuals from integrated-production farms and organic farms^75^. Our results raise similar issues about large-scale contamination, ubiquity of exposure within agricultural landscapes, and generalized exposure of wildlife irrespective of farming system. However, the number and concentrations of CUPs in small mammals here did not significantly differ between farming systems. Brodeur et al.^18^ highlighted that detection frequency of CUPs in two frog species in Argentina did not differ significantly among sites located along a gradient of distance to the nearest crop, with frogs sampled in places without pesticide treatment showing similar frequency of pesticide detection than frogs captured near crops. These results on terrestrial stages of amphibians echo the present findings. In our study, the lack of significant differences between farming systems, along with contrasted levels between habitats (i.e. the lowest or the highest numbers of compounds/concentrations in hedgerows), may be related to the mobility of small mammals and the spatial extent they exploit (the home-range has been reported to vary around 230–12,200 m^2^ for *Apodemus* mice and around 50–200 m^2^ for *Crocidura* shrews, and animals living in hedgerows are known to move to surrounding fields^82^). In agricultural landscapes, seasonal and annual variations in space use by small mammals have been evidenced, which translates for instance in great changes before and after harvest in terms of ranges, mobility and habitat preferences (e.g. cropped surfaces versus hedgerows and uncut set-aside patches)^83,84^. Furthermore, the mobility of preys of small mammals, which may move from adjacent fields to the areas exploited by small mammals, could also cause such a pattern of contamination of all animals whether their place of capture was treated or not. One may hypothesize in this context that processes of pesticide trophic transfer and bioaccumulation, and even potential biomagnification and biowidening, allow the circulation of some CUPs at relatively high levels in agricultural food webs. Although quantified at lower concentrations than in treated crops, presence of CUP have been shown to occur both in organic cultures and non-treated semi-natural habitats all along the year (i.e. at the beginning of crop season and post-harvest), and sometimes exceeding predicted environmental concentrations even in non-treated habitats^11,12^. Direct inputs of CUPs in non-treated patches from surrounding treatments might occur, for instance due to drift, volatilization, and run-off. The contamination of non-treated habitats might therefore be one of the reasons explaining the ubiquity of exposure of small mammals.

The results from Pelosi et al.^11^ highlighted the potential role of OF practices and semi-natural habitats to limit environmental and biota contamination by CUPs, thus providing refuges for wildlife within the landscape mosaic. However, these drivers seem insufficient to significantly buffer the exposure of animals at higher trophic levels and having a greater home-range. Our study showed that small mammals captured in various habitats within the agricultural mosaics were all contaminated, this at the scale of dozens of landscape windows over agroecosystems, which questions whether recovery and recolonization can be ensured. Our results suggest that the current surfaces under OF and mitigation measures through the presence of semi-natural habitats acting as refuges are deficient, or at least insufficient, at the present time to lead to concrete effects on exposure and thus potential unintentional negative effects of pesticides in terrestrial ecosystems.

By assessing the exposure of wildlife to pesticides in agricultural landscapes using hair samples, this study shed light on the extent and ubiquity of small mammal contamination by complex mixtures of both legacy and current fungicides, herbicides and insecticides. This study raises issues about pathways of wildlife exposure to pesticides, especially for CUPs which physico-chemical characteristics and use practices should normally limit bioaccumulation in the environment and in biota. Further investigation is needed to disentangle and characterize the role of various factors likely involved in the fate of legacy compounds: physico-chemical factors related to the compounds themselves (properties of the molecules such as *K*_*oa*_ and *K*_*ow*_, metabolization), environmental factors (soil properties, landscape, climate, use of other pesticides), and history of use (duration and frequency, quantity, surfaces).

Showing global contamination at a wildlife population level, our findings provide support for a better understanding of unintentional impacts of pesticides in agro-ecosystems, underlying that effects on biodiversity might not only be related to acute accidental poisoning and cascading resource depletion effects but also to chronic exposure. This constitutes a timely issue towards biodiversity conservation and also towards societal implications^6,85,86^.

Our results also raise questions about the ecotoxicological consequences of such an exposure to pesticide mixtures and provide useful data about the composition of the mixtures to be studied. This warrants further research about mixture toxicity and advocates for the development of toxicological reference values and critical body residues, especially for CUPs, as done for many other contaminants within the last decades^78,87^. This should be done fostering animal welfare and reduction of animal use, for instance by optimizing laboratory testing with assessment of both toxicity endpoints, including sublethal ones, and bioaccumulation at once and with refined methods to assess toxicological responses^88^, and focusing on non-invasive sampling through analysis of body fluids, faeces and hair or feathers^42^). The continuous improvements in analytical chemistry allowing multi-residue measurements in complex matrices and conceptual developments about exposome^78^ pave the way for further investigation about exposure to mixtures and “biowidening”. These issues deserve further attention and warrant further research about wildlife exposure to and contamination by CUP, which are currently sparsely studied and surveyed apart from the modelling approaches applied during marketing authorization and toxicovigilance.

From a regulatory perspective, our results question the efficacy of present pesticide management to protect wildlife, providing support to re-design risk assessment and mitigation measures, as called for in the recent literature^14,79,89^. Indeed, despite constantly tightening regulation and improvements in usage practices within the last decades, a pervasive exposure to dozens of CUPs is shown here in non-target vertebrates, which moreover questions whether the extent and spatial configuration of refuges is sufficient to ensure ecological recovery. This work further highlights the importance of monitoring initiatives to relevantly survey long-term and spatial trends of wildlife exposure to legacy, currently used and emerging compounds. This is a critical issue to detect unexpected events and to assess the efficiency of regulatory measures in order to achieve ecosystem protection goals^90^. Our survey on small mammals is one of the only large multi-residue screenings of CUPs in mammals. It can valuably contribute to phytopharmacovigilance and toxicovigilance schemes, and even to decision making.

## Methods

### Sampling

Free-ranging small mammals were sampled in two sites belonging to the Long-Term Socio-Ecological Research (LTSER) network in France: the Zone Atelier Arc Jurassien (ZAAJ, https://zaaj.univ-fcomte.fr/) in North-Eastern France, and the Zone Atelier Plaine & Val de Sèvre (ZAPVS, https://za-plaineetvaldesevre.com/) in mid-Western France. The farmland landscapes over the sites are dominated by cereal crops (mostly maize, wheat, barley, rye) and grasslands. The ZAPVS is a lowland arable farmland area, which covers 450 km^2^, including 450 farms and 13,000 agricultural fields^91^. The ZAAJ mostly covers the Jura Mountains, which are currently 44% woodland, showing diversified forests with meadow-woodland clearings and vast surfaces of grasslands. In this study we focused on the lowlands of the ZAAJ, where the arable farmland context is similar to the cereal cropland of the ZAPVS, although with greater surfaces of woodlands. Sampling sessions were conducted during springtime 2015 in ZAAJ (April–July), and springtime 2016 (May–June) in ZAPVS.

Over the site ZAAJ, sampling was conducted in five areas (two in organic farms, three in conventional farms), only in hedgerows, which were surrounded by maize (before and after seedling) and grasslands. In the ZAPVS, sampling was conducted in three different types of habitats in 60 agricultural landscapes of 1 km^2^, namely arable crops (mainly winter wheat and barley fields (*n* = 18) and four oilseed rape fields), grasslands, and hedgerows. For each habitat, animals were captured in areas treated and non-treated by pesticides: crops or grasslands cultivated under either conventional farming (CF) or organic farming (OF), respectively. Hedgerows are not supposed to be direct target for PPP treatment, but they may be unintentionally contaminated due to drift or run-off for instance, and the homerange of animals can overlap both the hedgerows and the surrounding open habitats. In order to investigate a potential buffering effect of organic farming within the home-range of animals on their exposure to PPP, we described the hedgerow surroundings with a “type of farming” variable refered to as “CF” when adjacent plots were cultivated under conventional farming only, and “OF” when at least one surrounding plot was cultivated under organic farming (i.e. two sides or one side of the hedgerow under OF).

Small mammals were captured using lines of non-lethal traps with dormitory filled with hay and baits^92^. Traplines were set during one to three nights depending on capture success (i.e. two or three nights in case of absence of rodent capture during the first night(s)). Traps were checked every day, rebaited and refilled as necessary, and reset (replaced with a new trap) in case of capture. Animals were held in traps until handling and released immediately after sampling. The duration of handling was kept as minimum (5–10 min) in order to limit stress. Welfare and distress were surveyed continuously by assessing visually behavior (alertness, responsiveness, grooming), breathing (chest/abdominal, rate) and tonus. Animals were handled following recommendations for manual manipulation of small mammals (i.e. tunnel-like picking up technique and tipped out backward, three-finger restraint technique, plastic tunnel-like restraint device during hair sampling). Individuals were released in the field at the location where captured after handling. Species were determined during handling of individuals alive based on morphometrics, and confirmed by molecular analyses in case of doubt (see below). Sex was not reported because such examination on living animals (1) does not provide certain evaluation depending on the species of concern and age/sexual maturity of individuals and seasonal variations in reproductive activity, and (2) would have implied longer handling. A sample of 50–100 mg of hair was taken by shaving over the posterior part of the back, wrapped in aluminium foil and stored in plastic zip-lock bags at room temperature during the field session and later at − 20 °C until analysis. For animals that did not survive to the capture, bodies were collected from traps and stored at − 20 °C until they were shaved for hair sampling in the laboratory.

Because *A. flavicollis* and *A. sylvaticus* are cryptic species (i.e. with close morphological characteristics, especially in juveniles), species identification of *Apodemus* mice was confirmed by molecular analyses (Supporting Methods). For that, a small piece of ear was sampled during handling (using medical clean and disinfected scissors, followed by disinfection of the ear) and stored by freezing (− 20 °C) until DNA extraction. This was realized for ZAAJ only since the species *A. flavicollis* is not present over the ZAPVS.

The final sample size resulted in 93 individuals for which hair samples were analyzed, with 77 and 16 individuals from ZAPVS and ZAAJ sites, respectively (Table 3). In site ZAPVS, 63 shrews *C. russula* were captured in three different habitats (22, seven and 34 individuals in cereal crops, grasslands and hedgerows, respectively) and 14 wood mice *A. sylvaticus* from the same hedgerows as shrews. In ZAAJ site, eight wood mice *A. sylvaticus* and eight yellow-necked mice *A. flavicollis* were captured in hedgerows from ZAAJ. A total of 26 individuals were trapped in plots under OF or in hedgerows surrounded by field(s) under OF.

We confirm that all methods were carried out in accordance with relevant guidelines and regulations. All experimental protocols were approved by a named institutional and licensing committee as detailed hereafter. The experimentation was performed under the authorization of the French National Ethical Committee (Project APAFIS N°5340) by skilled and experienced investigators from Chrono-environnement research department (EU0592), following directive 2010/63/EU on the protection of animals used for scientific purposes. All precautions to limit as much as possible stress and deleterious effects on animals were taken. Sampling and handling of small mammals was conducted under the supervision and with the participation of people authorized for animal experimentation (R.S. and C.F.), highly experienced in small mammal capture and handling, and using appropriate and authorized (EU0592) facilities (vehicle equipped with mobile anaesthesia unit (UNIVENTOR 400 unit for Isoflurane anaesthesia), and all materials and equipment required for animal welfare). We hereby confirm that the study is reported in accordance with ARRIVE guidelines.

### Chemical analyses

No exclusion criteria were applied, all the 93 individual samples were included in the set of analyses. The samples were labeled in order that investigators were unaware of the “treatment” of animals (e.g. species, habitat, or type of farming at capture location). A multi-residue analysis of 140 chemicals, parent pesticides and metabolites of banned or restricted (67) and currently used (73) pesticides (Tables 1 and 2), was performed. The classification as BRP or CUP was mainly based on regulatory status thus on their potential for current actual use. We also directed the classification to consider separately the legacy persistent and bioaccumulative pesticides versus the new generations of pesticides designed with the purpose to limit unintentional effects and submitted to thorough environmental risk assessment process before marketing authorization. The compounds classified as BRPs were thus legacy chemicals which have been banned or restricted to biocide or veterinary product use (e.g. fipronil) in Europe and/or France. Their presence in the environment and in biota should therefore not be caused by current or recent (i.e. around 3–5 years, see below) use as pesticides, but only due to environmental persistence, unless illegal use. The compounds classified as CUPs were currently used in agriculture since they were registered and authorized for pesticide use in Europe and/or France at the time of our sampling sessions. A few chemicals under ban or restriction for use as pesticides in the 2010’s have been here classified as CUPs, although not officially authorized in 2016. This because, after ban, pesticides can be used purposedly during a few years until stock depletion or remained authorized for specific usages in absence of alternative practices (e.g. specific crops/specific pest). In addition, with regards to chemical properties and ERA processes as well as to usages in other countries, they were more comparable to CUP rather than to legacy compounds. This is the case notably for carbendazim and dimethoate, and some pyrethroids like permethrine and cyfluthrine. The metabolites were classified as BRPs or CUPs according to the status of their parent compounds. The classification was complex for some non-specific metabolites of pyrethroids and organophosphates since they could originate from parent pesticides banned or currently used (e.g. Supplementary Fig. S2). Since several pyrethroids were still currently used and some others banned within the very last years and potentially still in use by usage derogation, it was choosen to classify the metabolites in the group of CUPs. Contrarily, since the majority of organophosphorous pesticides were banned, dialkyl phosphates (DAPs) that are non-specific metabolites were classified as BRPs (Supplementary Fig. S2).

Detailed use of each CUP in terms of type and timing of application and of quantities in the cultivated fields in ZAPVS and ZAAJ were not available. The yearly quantities sold in 2016 at the regional level were available in the national registry BNVD^58^, and were considered as a proxy of use although imperfect and undetailed both spatially and temporally.

For CUPs, 66 parent molecules and seven metabolites were screened (25 belonging to type of usage as fungicides, 23 to herbicides and 25 to insecticides). For BRPs, 39 parent molecules and 28 metabolites were screened (five belonging to type of usage as fungicides, 19 to herbicides and 43 to insecticides). Chemical analyses were performed according to a method previously developed and validated for the analysis of pesticides in human and animal hair^48,93^. Briefly, hair samples were decontaminated with sodium dodecyl sulfate (Sigman-Aldrich; CAS: 151-21-3) and methanol (Biosolve; CAS: 67-56-1) according to a previously validated protocol in order to remove chemicals possibly deposited on hair surface^94^. After drying, samples were pulverized using a ball mill (RETSCH, MM200) and 50 mg of hair powder were used for the extraction with water:acetonitrile (20/80) (Biosolve; CAS: 75-05-8) after addition of stable isotope labelled analogues as internal standards (information available upon request to the authors). Extracts were then splitted and evaporated under nitrogen and reconstituted in adequate solvents according to the method used for analysis (GC–MS/MS and LC–MS/MS). The analytical method was presented in details in previous articles^45,93^. The extracts were then analyzed with GC–MS/MS (Agilent, 7890A, MS 7000A) and LC–MS/MS (Waters, Acquity UPLC-Xevo TQ-S). In each analytical run, quality controls were analyzed along with the field samples. The quality controls consisted of one blank and eight supplemented controls at different concentrations ranging from 0.5 to 100 ng/g. The blanks allowed to confirm the absence of cross-contamination and the supplemented controls to monitor any possible drift in the analytical response. Limits of quantification (LOQ) and lowest detected values (no data in case of absence of any detection) are presented in Tables 1 and 2. All detected concentration values were included as continuous data, including values below the LOQ and the limit of detection (LOD). The LOD was set at the lowest detected value. Several methods have been described to handle data below the LOQ, with various advantages or disavantages and relevance depending on conditions for application such as sample size and data distribution. It has, however, been shown that the use of concentrations below the LOQ exhibited better efficiency over recognized established methods in terms of bias and precision and was feasible and preferable to the absence of data^95^. For further statistical analyses (see below), the concentration value was set at 0 for data under the lowest detected value (i.e. LOD).

### Data analysis and statistics

All analyses were performed using R (version 3.3.1), with the additional packages “partykit” “pgirmess”, “strucchange”, and “vegan”.

We first described general patterns of detection and quantification of both BRPs and CUPs considering the number and percentage of compounds found (1) relative to the set of compounds screened and (2) relative to their occurrence in small mammals. The occurrence and concentrations of CUPs in small mammals were compared to their putative use by checking for correlation between (1) number of detections or (2) number of quantifications above 10 ng/g in small mammal individuals, and the quantity of each corresponding pesticide sold in 2016 in Deux-Sèvres (location of site ZAPVS where most of small mammals have been captured). The metabolites of pyrethroids were not included since they could not be related to specific corresponding pesticides. The quantities of chlorpyriphos-ethyl and chlorpyriphos-methyl were summed and related to the detection/quantification of the metabolite TCPy. Similarly, the quantities of mecoprop (mcpp) and mecoprop-p (mcpp-p) were summed. The non-parametric Spearman’s rank correlation test was used to analyze these relationships.

We then investigated the patterns of contamination by considering (1) the occurrence of chemicals (number by individual, for all the compounds and for compounds pooled according to type of PPP use: “fungicides”, “herbicides”, “insecticides”) and (2) the sum of concentrations (by individual, molecules pooled according to type of PPP use: “fungicides”, “herbicides”, “insecticides”). The analyses were conducted for BRPs and CUPs separately. Differences between mice and shrews (data on mice *A. sylvaticus* and shrews *C. russula* captured at same location in ZAPVS) and between sampling zones (data on mice *Apodemus* from ZAPVS and ZAAJ) were each tested using the *t-test* or the *Wilcoxon–Mann–Whitney test* when necessary (when assumptions regarding the normality and homoscedasticity of variances were not respected). The differences between the types of habitat (data on shrews *C. russula* from ZAPVS), were analyzed using *ANOVA* or alternatively *Kruskal–Wallis test*. *Post-hoc* tests (*Tukey’s HSD method* after *ANOVA* or multiple comparison test after *Kruskal–Wallis* test) were used to investigate pair-comparisons when relevant. Differences between types of farming OF and CF were analyzed for each subset of data (i.e. shrews from ZAPVS, mice from ZAAJ and ZAPVS, or shrews and mice at the same location in ZAPVS) using the *t-test* or the *Wilcoxon–Mann–Whitney test* when necessary.

Redundancy multivariate analyses (RDA) were then used to analyze the profiles of molecules according to each of the following factors: type of habitat (cereal, grassland or hedgerow; data shrews *C. russula* from ZAPVS), or species (*A. sylvaticus* or *C. russula*; data of mice and shrews captured at same location in ZAPVS)*,* or zone (ZAAJ or ZAPVS; data of *Apodemus* mice from ZAPVS and ZAAJ) or type of farming (OF and CF). Analyses were conducted separately for BRPS and CUPs. The pesticides that were not detected were discarded from the respective datasets. Response data were log-transformed (log10*x* + 0.1) to meet assumptions of distribution normality and linearity. In order to investigate the specific significance of the factors, the “best” models were selected based on the significance of the *F-statistics* associated with all variables (tested using permutation tests) and Akaike Information Criterion (AIC) using the function “ordistep” in “vegan”^96^. Adjusted R-squared were computed according to Borcard et al.

As a final step, multivariate conditional inference trees^97–99^ on the whole dataset were used to identify the hierarchy of factors in shaping the profiles of contamination and the most discriminant pesticides in such profiles, using the same explanatory factors as described above (type of habitat, species, zone, and type of farming). Conditional inference trees were computed separately for BRPS and CUPs, using all molecules as response variables except the ones that were never detected. Trees were computed using the “ctree” function in “partykit” with the following control options: the statistical test was set at “maximum” since we test multi-level categorical factors, and “Bonferroni correction” was applied in the test to avoid *p-value* bias in resampling approach.

## Supplementary Information

Supplementary Information 1.

Supplementary Information 2.

## Data Availability

The full dataset is available in Supplementary Information files.

## References

[1] Rattner BA (2009). History of wildlife toxicology. Ecotoxicology.

[2] European Parliament and Council of the European Union. *Directive 2009/128/EC of the European Parliament and of the Council of 21 October 2009 establishing a framework for Community action to achieve the sustainable use of pesticides (Text with EEA relevance)*. 71–86 (2009).

[3] McGrath PF (2014). Politics meets Science: The case of neonicotinoid insecticides in Europe. Surveys Perspect. Integr. Environ. Society.

[4] EFSA. How Europe ensures pesticides are safe. https://www.efsa.europa.eu/en/interactive-pages/pesticides-authorisation/PesticidesAuthorisation (2020). Accessed 13 February 2020.

[5] US EPA. Pesticides-Pesticide Regulation-Protecting Health & the Environment. https://www.epa.gov/pesticides (2020). Accessed 13 February 2020.

[6] Bernhardt ES, Rosi EJ, Gessner MO (2017). Synthetic chemicals as agents of global change. Front. Ecol. Environ..

[7] FAO. Integrated Production and Pest Management Programme in Africa. *The Integrated Production and Pest Management (IPPM) Programme* http://www.fao.org/agriculture/ippm/programme/en/ (2022). Accessed 27 April 2022.

[8] Rohr JR (2019). Emerging human infectious diseases and the links to global food production. Nat. Sustain..

[9] Sabatier P (2014). Long-term relationships among pesticide applications, mobility, and soil erosion in a vineyard watershed. Proc. Natl. Acad. Sci..

[10] Hvězdová M (2018). Currently and recently used pesticides in Central European arable soils. Sci. Total Environ..

[11] Pelosi C (2021). Residues of currently used pesticides in soils and earthworms: A silent threat?. Agr. Ecosyst. Environ..

[12] Geissen V (2021). Cocktails of pesticide residues in conventional and organic farming systems in Europe—Legacy of the past and turning point for the future. Environ. Pollut..

[13] Sabzevari S, Hofman J (2022). A worldwide review of currently used pesticides’ monitoring in agricultural soils. Sci. Total Environ..

[14] Brühl CA, Zaller JG (2019). Biodiversity decline as a consequence of an inappropriate environmental risk assessment of pesticides. Front. Environ. Sci..

[15] Gómez-Ramírez P (2014). An overview of existing raptor contaminant monitoring activities in Europe. Environ. Int..

[16] Douglas MR, Rohr JR, Tooker JF (2015). Neonicotinoid insecticide travels through a soil food chain, disrupting biological control of non-target pests and decreasing soya bean yield. J. Appl. Ecol..

[17] Millot F (2017). Field evidence of bird poisonings by imidacloprid-treated seeds: A review of incidents reported by the French SAGIR network from 1995 to 2014. Environ. Sci. Pollut. Res..

[18] Brodeur JC (2022). Concentration of current-use pesticides in frogs from the Pampa region and correlation of a mixture toxicity index with biological effects. Environ. Res..

[19] Kuzukiran O (2021). Multiresidues of environmental contaminants in bats from Turkey. Chemosphere.

[20] EUROPEAN COMMISSION. *Technical Guidance Document on risk assessment in support of Commission Directive 93/67/EEC on risk assessment for new notified substances, Commission Regulation (EC) No 1488/94 on risk assessment for existing substances, Directive 98/8/EC of the European Parliament and of the Council concerning the placing of biocidal products on the market. Part II*. (2003).

[21] EFSA (2008). Scientific Opinion of the Panel on Plant protection products and their Residues (PPR) on the science behind the guidance document on risk assessment for birds and mammals. EFSA J..

[22] EFSA (2009). Guidance of EFSA—Risk assessment for birds and mammals. EFSA J..

[23] Barber I, Tarrant KA, Thompson HM (2003). Exposure of small mammals, in particular the wood mouse *Apodemus sylvaticus*, to pesticide seed treatments. Environ. Toxicol. Chem..

[24] Block EK, Lacher TE, Brewer LW, Cobb GP, Kendall RJ (1999). Population responses of Peromyscus resident in Iowa cornfields treated with the organophosphorus pesticide COUNTER (R). Ecotoxicology.

[25] Guitart R (2010). Animal poisoning in Europe. Part 3: Wildlife. Vet. J..

[26] Prosser RS, Anderson JC, Hanson ML, Solomon KR, Sibley PK (2016). Indirect effects of herbicides on biota in terrestrial edge-of-field habitats: A critical review of the literature. Agric. Ecosyst. Environ..

[27] Delibes-Mateos M, Smith AT, Slobodchikoff CN, Swenson JE (2011). The paradox of keystone species persecuted as pests: A call for the conservation of abundant small mammals in their native range. Biol. Cons..

[28] Gibbons, J. W. *The management of amphibians, reptiles, and small mammals in North America: Need for an environmental attitude adjustment*. 4–10 (1988).

[29] Gutierrez-Arellano C, Mulligan M (2018). A review of regulation ecosystem services and disservices from faunal populations and potential impacts of agriculturalisation on their provision, globally. Nat. Conserv. Bulgaria..

[30] Martin BG (2003). The role of small ground-foraging mammals in topsoil health and biodiversity: Implications to management and restoration. Ecol. Manag. Restor..

[31] Sieg, C. H. Small mammals: Pests or vital components of the ecosystem. in 88–92 (1987).

[32] Sullivan TP, Sullivan DS (2009). Are linear habitats in agrarian landscapes source areas of beneficial or pest rodents?. Agric. Ecosyst. Environ..

[33] Tschumi M, Ekroos J, Hjort C, Smith HG, Birkhofer K (2018). Predation-mediated ecosystem services and disservices in agricultural landscapes. Ecol. Appl..

[34] Coeurdassier, M., Fritsch, C., Jacquot, M., van den Brink, N. W. & Giraudoux, P. Spatial Dimensions of the Risks of Rodenticide Use to Non-target Small Mammals and Applications in Spatially Explicit Risk Modeling. in *Anticoagulant Rodenticides and Wildlife* (eds. van den Brink, N. W., Elliott, J. E., Shore, R. F. & Rattner, B. A.) vol. 5, 195–227 (Springer International Publishing, 2018).

[35] Talmage S, Walton B (1991). Small mammals as monitors of environmental contaminants. Rev. Environ. Contaminants Toxicol..

[36] Baudrot V, Fritsch C, Perasso A, Banerjee M, Raoul F (2018). Effects of contaminants and trophic cascade regulation on food chain stability: Application to cadmium soil pollution on small mammals—Raptor systems. Ecol. Modell..

[37] Roos S (2021). Annual abundance of common Kestrels (Falco tinnunculus) is negatively associated with second generation anticoagulant rodenticides. Ecotoxicology.

[38] International Union for the Conservation of Nature. The IUCN Red List of Threatened Species. Version 2020-1. (2020).

[39] Gobas FA (2016). Review of existing terrestrial bioaccumulation models and terrestrial bioaccumulation modeling needs for organic chemicals. Integr. Environ. Assess. Manag..

[40] Kelly BC, Gobas FAPC (2003). An arctic terrestrial food-chain bioaccumulation model for persistent organic pollutants. Environ. Sci. Technol..

[41] Li, Z. J. Spatiotemporal pattern models for bioaccumulation of pesticides in herbivores: An approximation theory for North American white-tailed deer. *Sci. Total Environ*. **737**, (2020). 10.1016/j.scitotenv.2020.140271 32783856

[42] Zemanova, M. A. Towards more compassionate wildlife research through the 3Rs principles: Moving from invasive to non-invasive methods. *Wildl. Biol*. **2020**, (2020).

[43] Espín S (2016). Tracking pan-continental trends in environmental contamination using sentinel raptors—What types of samples should we use?. Ecotoxicology.

[44] Tête N (2014). Hair as a noninvasive tool for risk assessment: Do the concentrations of cadmium and lead in the hair of wood mice (Apodemus sylvaticus) reflect internal concentrations?. Ecotoxicol. Environ. Saf..

[45] Appenzeller BMR (2017). Hair analysis for the biomonitoring of pesticide exposure: Comparison with blood and urine in a rat model. Arch. Toxicol..

[46] Fäys F (2021). Biomonitoring of fast-elimination endocrine disruptors—Results from a 6-month follow up on human volunteers with repeated urine and hair collection. Sci. Total Environ..

[47] Appenzeller BMR, Tsatsakis AM (2012). Hair analysis for biomonitoring of environmental and occupational exposure to organic pollutants: State of the art, critical review and future needs. Toxicol. Lett..

[48] Chata C, Hardy E, Grova N, Appenzeller BMR (2016). Influence of pesticide physicochemical properties on the association between plasma and hair concentration. Analyt. Bioanalyt. Chem..

[49] Botías C, David A, Hill EM, Goulson D (2016). Contamination of wild plants near neonicotinoid seed-treated crops, and implications for non-target insects. Sci. Total Environ..

[50] Lennon, R. J. *et al.* From seeds to plasma: Confirmed exposure of multiple farmland bird species to clothianidin during sowing of winter cereals. *Sci. Total Environ*. **723**, (2020). 10.1016/j.scitotenv.2020.138056 32224397

[51] Wong F (2021). Time trends of persistent organic pollutants (POPs) and Chemicals of Emerging Arctic Concern (CEAC) in Arctic air from 25 years of monitoring. Sci. Total Environ..

[52] Badry A, Schenke D, Treu G, Krone O (2021). Linking landscape composition and biological factors with exposure levels of rodenticides and agrochemicals in avian apex predators from Germany. Environ. Res..

[53] Di Blasio, A. *et al.* Local context and environment as risk factors for acute poisoning in animals in northwest Italy. *Sci. Total Environ*. **709**, (2020). 10.1016/j.scitotenv.2019.136016 31905591

[54] Ntemiri, K. *et al.* Animal mortality and illegal poison bait use in Greece. *Environ. Monit. Assessment*. **190**, (2018). 10.1007/s10661-018-6838-5 30046915

[55] Kitowski I, Łopucki R, Stachniuk A, Fornal E (2021). Banned pesticide still poisoning EU raptors. Science.

[56] Mateo-Tomas P, Olea PP, Minguez E, Mateo R, Vinuela J (2020). Direct evidence of poison-driven widespread population decline in a wild vertebrate. Proc. Natl. Acad. Sci. USA..

[57] Plaza PI, Martinez-Lopez E, Lambertucci SA (2019). The perfect threat: Pesticides and vultures. Sci. Total Environ..

[58] BNVD. Banque Nationale des ventes de produits phytopharmaceutiques par les Distributeurs agréés. https://bnvd.ineris.fr/. https://bnvd.ineris.fr/ (2022). Accessed 02 February 2022.

[59] Kelly BC, Ikonomou MG, Blair JD, Morin AE, Gobas FAPC (2007). Food web-specific biomagnification of persistent organic pollutants. Science.

[60] Butet A, Delettre YR (2011). Diet differentiation between European arvicoline and murine rodents. Acta Theriol..

[61] Singer MS, Bernays EA (2003). Understanding omnivory needs a behavioral perspective. Ecology.

[62] Smith PN (2007). Contaminant exposure in terrestrial vertebrates. Environ. Pollut..

[63] European Food Safety Authority (EFSA). Risk Assessment for Birds and Mammals—Revision of Guidance Document under Council Directive 91/414/EEC (SANCO/4145/2000—Final of 25 September 2002)—Scientific Opinion of the Panel on Plant protection products and their Residues (PPR) on the Science behind the Guidance Document on Risk Assessment for birds and mammals. *EFS2* **6**, (2008). 10.2903/j.efsa.2008.734 PMC10193663 37213850

[64] MacDonald AM, Jardine CM, Thomas PJ, Nemeth NM (2018). Neonicotinoid detection in wild turkeys (Meleagris gallopavo silvestris) in Ontario, Canada. Environ. Sci. Pollut. Res..

[65] Cobb GP, Mellott R, Brewer LW, Bens CM, Kendall RJ (2000). Diazinon dissipation from vegetation, occurrence in earthworms, and presence in avian gastrointestinal tracts collected from apple orchards following D-z-n((R)) 50W application. Environ. Toxicol. Chem..

[66] Humann-Guilleminot S (2021). Contamination by neonicotinoid insecticides in barn owls (Tyto alba) and Alpine swifts (Tachymarptis melba). Sci. Total Environ..

[67] Poisson M (2021). Assessing pesticides exposure effects on the reproductive performance of a declining aerial insectivore. Ecol. Appl..

[68] Graves EE (2019). Analysis of insecticide exposure in California hummingbirds using liquid chromatography-mass spectrometry. Environ. Sci. Pollut. Res..

[69] Mayer, M., Duan, X. D., Sunde, P. & Topping, C. J. European hares do not avoid newly pesticide-sprayed fields: Overspray as unnoticed pathway of pesticide exposure. *Sci. Total Environ*. **715**, (2020). 10.1016/j.scitotenv.2020.136977 32014783

[70] Bro E, Devillers J, Millot F, Decors A (2016). Residues of plant protection products in grey partridge eggs in French cereal ecosystems. Environ. Sci. Pollut. Res..

[71] Rial-Berriel C (2021). A method scope extension for the simultaneous analysis of POPs, current-use and banned pesticides, rodenticides, and pharmaceuticals in liver. Application to food safety and biomonitoring. Toxics.

[72] Kaczyński P (2021). Impact of broad-spectrum pesticides used in the agricultural and forestry sector on the pesticide profile in wild boar, roe deer and deer and risk assessment for venison consumers. Sci. Total Environ..

[73] Smalling KL (2015). Pesticide concentrations in frog tissue and wetland habitats in a landscape dominated by agriculture. Sci. Total Environ..

[74] Bishop, C. A. *et al.* Determination of neonicotinoids and butenolide residues in avian and insect pollinators and their ambient environment in Western Canada (2017, 2018). *Sci. Total Environ*. **737**, (2020). 10.1016/j.scitotenv.2020.139386 32563110

[75] Humann-Guilleminot S (2019). A large-scale survey of house sparrows feathers reveals ubiquitous presence of neonicotinoids in farmlands. Sci. Total Environ..

[76] Byholm P, Mäkeläinen S, Santangeli A, Goulson D (2018). First evidence of neonicotinoid residues in a long-distance migratory raptor, the European honey buzzard (Pernis apivorus). Sci. Total Environ..

[77] Bertrand, C. *et al.* Assessing the impact of farming practices and landscape heterogeneity on ground beetles’ exposure to pesticides. (2018).

[78] Scholz S (2022). The eco-exposome concept: Supporting an integrated assessment of mixtures of environmental chemicals. Environ. Toxicol. Chem..

[79] Weisner O (2021). Risk from pesticide mixtures—The gap between risk assessment and reality. Sci. Total Environ..

[80] Gibbons D, Morrissey C, Mineau P (2015). A review of the direct and indirect effects of neonicotinoids and fipronil on vertebrate wildlife. Environ. Sci. Pollut. Res..

[81] Mineau, P. & Callaghan, C. *Neonicotinoid insecticides and bats: An assessment of the direct and indirect risks*. 87 (2018).

[82] Wijnhoven S, Van Der Velde G, Leuven R, Smits AJM (2005). Flooding ecology of voles, mice and shrews: The importance of geomorphological and vegetational heterogeneity in river floodplains. Acta Theriol..

[83] Stradiotto A (2009). Spatial organization of the yellow-necked mouse: Effects of density and resource availability. J. Mammal..

[84] Tattersall FH, Macdonald DW, Hart BJ, Manley WJ, Feber RE (2001). Habitat use by wood mice (Apodemus sylvaticus) in a changeable arable landscape. J. Zool..

[85] Groh K, vom Berg C, Schirmer K, Tlili A (2022). Anthropogenic chemicals as underestimated drivers of biodiversity loss: Scientific and societal implications. Environ. Sci. Technol..

[86] Persson L (2022). Outside the safe operating space of the planetary boundary for novel entities. Environ. Sci. Technol..

[87] Beyer, W. N., Heinz, G. H. & Redmon-Norwood, A. W. *Environmental contaminants in wildflife. Interpreting tissue concentrations*. (Society of Environmental Toxicology and Chemistry (SETAC), 1996).

[88] Arck PC (2019). When 3 Rs meet a forth R: Replacement, reduction and refinement of animals in research on reproduction. J. Reprod. Immunol..

[89] Topping CJ, Aldrich A, Berny P (2020). Overhaul environmental risk assessment for pesticides. Science.

[90] Vijver MG (2017). Postregistration monitoring of pesticides is urgently required to protect ecosystems. Environ. Toxicol. Chem..

[91] Bretagnolle V (2018). Towards sustainable and multifunctional agriculture in farmland landscapes: Lessons from the integrative approach of a French LTSER platform. Sci. Total Environ..

[92] Le Quilliec, P. & Croci, S. Piégeage de micromammifères : une nouvelle boîte-dortoir pour le piège non vulnérant INRA. *Cahier des Techniques de l’INRA* 125–128 (2006).

[93] Béranger R (2018). Multiple pesticide analysis in hair samples of pregnant French women: Results from the ELFE national birth cohort. Environ. Int..

[94] Duca R-C, Hardy E, Salquèbre G, Appenzeller BMR (2014). Hair decontamination procedure prior to multi-class pesticide analysis: Hair decontamination. Drug Test. Anal..

[95] Keizer, R. J. *et al.* Incorporation of concentration data below the limit of quantification in population pharmacokinetic analyses. *Pharmacol. Res. Perspect*. **3**, (2015). 10.1002/prp2.131 PMC4448983 26038706

[96] Borcard D, Gillet F, Legendre L (2011). Numerical Ecology with R.

[97] De’ath G, Fabricius KE (2000). Classification and regression trees: A powerful yet simple technique for ecological data analysis. Ecology.

[98] Hothorn T, Hornik K, Zeileis A (2006). Unbiased recursive partitioning: A conditional inference framework. J. Comput. Graph. Stat..

[99] Hothorn T, Zeileis A (2015). Partykit: A modular toolkit for recursive partytioning in R. J. Mach. Learn. Res..

