# Supplementary material for: Pervasive exposure of wild small mammals to legacy and currently used pesticide mixtures in arable landscapes
Source: Sci Rep. 2022 Sep 23;12:15904. doi: 10.1038/s41598-022-19959-y (PMC9508241; doi:10.1038/s41598-022-19959-y)

Pervasive exposure of wild small mammals to legacy and currently used pesticide mixtures in arable landscapes

Authors and affiliations

Clémentine Fritsch [1,2] * ¤, Brice Appenzeller [3] ¤, Louisiane Burkart [1], Michael Coeurdassier [1], Renaud Scheifler [1], Francis Raoul [1], Vincent Driget [1], Thibaut Powolny [1], Candice Gagnaison [1], Dominique Rieffel [1], Eve Afonso [1], Anne-Claude Goydadin [1], Emilie M Hardy [3], Paul Palazzi [3], Charline Schaeffer [3], Sabrina Gaba [4,5], Vincent Bretagnolle [4,5], Colette Bertrand [6], Céline Pelosi [6,7]

(1) UMR 6249 Chrono-environnement CNRS - Université de Franche-Comté, 16 route de Gray 25030 Besançon cedex

(2) LTSER « Zone Atelier Arc Jurassien », 25030 Besançon cedex, France

(3) Luxembourg Institute of Health, Dpt of Population Health, 29 Rue Henri Koch, 4354 Esch-sur Alzette, Luxembourg

(4) UMR 7372 CEBC, CNRS – La Rochelle Université, USC INRAE, 405 route de Prissé la Charrière, 79360 Villiers-en-Bois, France

(5) LTSER « Zone Atelier Plaine & Val De Sèvre », 79360 Beauvoir Sur Niort, France

(6) UMR 1402 EcoSys, INRAE – AgroParisTech – Université Paris-Saclay, RD 10 Route de St Cyr, 78026 Versailles Cedex, France

(7) UMR EMMAH, INRAE - Avignon Université, F-84000, Avignon, France

¤ These authors contributed equally to this work

* Corresponding Author:

Dr Clémentine Fritsch

UMR 6249 Chrono-environnement

CNRS - Université de Franche-Comté

16 route de Gray, F-25030 Besançon cedex

clementine.fritsch@univ-fcomte.fr

Supplementary Information

List of appendices

Supplementary Table S1. Results on statistical differences between habitats, species, sites and type of farming in the number of pesticide molecules detected by individual. 3

Supplementary Table S2. Results on statistical differences between habitats, species and sites in the concentrations of pesticides quantified by individual. 4

Supplementary Table S3. Extract of sales of pesticides in 2016 in Deux-Sèvres (location of site ZAPVS where most of small mammals have been captured) ordered according to the quantity sold. 5

Supplementary Methods. Molecular identification of small mammal species 7

Supplementary Figure S1. Relationships between A) number of detection in hair of small mammals or B) number of quantification above 10 ng/g in hair of small mammals and the quantity of each corresponding pesticide sold in 2016 in Deux-Sèvres (location of site ZAPVS where most of small mammals have been captured) with results of non-parametric tests of correlation. 8

Supplementary Figure S2. Correspondence between parent organophosphorous pesticides and their metabolites. 9

Supplementary Table S1. Results on statistical differences between habitats, species, sites and type of farming in the number of pesticide molecules detected by individual.

The abbrevations of factor levels are: conventional farming “CF”, organic farming “OF”, wood mouse *Apodemus sylvaticus* “Apsy”, and shrews *Crocidura russula* “Cru”. The statistical tests performed are referred to as ANOVA “AOV”, Kruskal-Wallis “KW”, t-test “*t*”, and Wilcoxon-Mann-Whitney “MN”. Statical significance is indicated with the following signs: “ns” non-significant *p-value* > 0.05 (in case the p-value was close to significance the exact *p-value* is provided), * for 0.05 ≥ *p-value* > 0.01, ** for 0.01 ≥ *p-value* > 0.001, *** *p-value* < 0.001. Significant differences between levels are indicated by uppercase letters, levels sharing the same letter did not differed statistically and data showing significant differences are written in bold.

Dataset	Factors levels	Number of Molecules						Number of Fungicides						Number of Herbicides						Number of Insecticides					
		Min	Max	Med	Mean		Test	Min	Max	Med	Mean		Test	Min	Max	Med	Mean		Test	Min	Max	Med	Mean		Test
**BRPs**																									
Shrews from	**Habitat**																								
ZAPVS in three	Cereal	15	26	18.5	18.8		AOV	**1**	**2**	**2**	**1.6**	a	**KW**	**3**	**11**	**4**	**4.6**	a	**KW**	**10**	**18**	**12**	**12.6**	a	**KW**
habitats	Grassland	17	22	18.0	18.6		ns	**1**	**2**	**1**	**1.1**	a	***	**4**	**5**	**5**	**4.9**	ab	***	**11**	**16**	**13**	**12.6**	a	***
(*n*=63)	Hedgerow	14	22	18.0	18.3			**0**	**3**	**3**	**2.3**	b		**3**	**7**	**6**	**5.6**	b		**8**	**15**	**10**	**10.3**	b	
	**Farming**																								
	CF	14	26	18	18.6		*t*	0	3	2	1.9		MN	3	**11**	**5**	**5.1**	a	**MN**	8	18	11	11.6		MN
	OF	16	21	18	18.2		ns	1	3	2	2.1		ns	4	**7**	**5**	**5.4**	b	**	9	14	11	10.7		ns
Shrews and	**Species**																								
wood mice	Apsy	**13**	**19**	**15.0**	**15.1**	a	*t*	**1**	**2**	**1**	**1.1**	a	**MN**	**3**	**4**	**3**	**3.4**	a	**MN**	9	14	10	10.6		*t*
from ZAPVS in	Crru	**16**	**21**	**18.5**	**18.8**	b	***	**0**	**3**	**3**	**2.5**	b	***	**4**	**7**	**6**	**6.0**	b	***	9	12	10	10.3		ns
hedgerows	**Farming**																								
(*n*=28)	CF	13	21	17.0	16.8		*t*	0	3	1.5	1.8		MN	3	7	4.5	4.7		*t*	9	14	10	10.3		*t*
	OF	14	21	16.5	17.4		ns	1	3	1.5	1.9		ns	3	7	4.0	4.8		ns	10	12	11	10.8		ns
Wild mice	**Zone**																								
from the two	ZAAJ	13	19	15	15.4		*t*	0	2	1	1.0		MN	3	5	4	4.2	a	MN	9	13	10	10.2		MN
zones in	ZAPVS	13	19	15	15.1		ns	1	2	1	1.1		ns	3	4	3	3.4	b	**	9	14	10	10.6		ns
hedgerows	**Farming**																								
(n=30)	CF	13	19	15	15.3		*t*	0	2	1	1.2		MN	3	5	4	3.7		MN	9	14	10	10.5		MN
	OF	13	19	14	15.1		ns	0	2	1	0.8		ns	3	5	4	4.1		ns	9	12	10	10.2		ns
**CUPs**																									
Shrews from	**Habitat**																								
ZAPVS in three	Cereal	**24**	**41**	**33**	**33.3**	ab	**AOV**	**6**	**13**	**11**	**10.3**	a	**KW**	11	17	14.5	14.5		KW	6	12	8.5	8.6		KW
habitats	Grassland	**24**	**38**	**28**	**30.6**	a	*	**5**	**10**	**9**	**7.7**	a	***	12	17	13,0	13.7		ns	6	12	10.0	9.1		ns
(*n*=63)	Hedgerow	**29**	**35**	**35**	**34.9**	b		**10**	**15**	**13**	**12.8**	b		11	16	15,0	14.2			6	15	8.0	7.8		
	**Farming**																								
	CF	24	41	34	33.7		*t*	5	15	11	11.2		MN	11	17	15	14.3		MN	6	15	8.0	8.3		MN
	OF	24	40	34.5	34.1		ns	6	15	12	11.9		ns	12	17	14	14.1		ns	6	12	8.0	8.2		*p*=0.05
Shrews and	**Species**																								
wood mice	Apsy	**22**	**36**	**29.0**	**28.5**	a	**MN**	**5**	**12**	**7.5**	**7.5**	a	*t*	**10**	**14**	**12**	**12.0**	a	*t*	5	15	8.5	9.0		MN
from ZAPVS in	Crru	**31**	**40**	**34.5**	**34.6**	b	***	**10**	**15**	**12.5**	**12.6**	b	***	**13**	**16**	**15**	**14.4**	b	***	6	10	8.0	7.9		ns
hedgerows	**Farming**																								
(*n*=28)	CF	22	38	33	32.0		*t*	5	14	11	10.1		*t*	10	16	13.0	13.3		*t*	6	15	8	8.7		*t*
	OF	24	40	32	30.9		ns	5	15	10	9.9		ns	11	16	12.5	13.1		ns	5	13	7	7.9		ns
Wild mice	**Zone**																								
from the two	ZAAJ	**18**	**28**	**23**	**22.8**	a	**MN**	4	11	7.0	7.1		*t*	**7**	**12**	**10**	**10.3**	a	*t*	**3**	**12**	**5.0**	**5.3**	a	*t*
zones in	ZAPVS	**22**	**36**	**29**	**28.5**	b	**	5	12	7.5	7.5		ns	**10**	**14**	**12**	**12.0**	b	***	**5**	**15**	**8.5**	**9.0**	b	**
hedgerows	**Farming**																								
(*n*=30)	CF	18	36	25.5	26.35		MN	4	12	7.5	7.6		*t*	7	14	11.0	11.3		*t*	3	15	7	7.5		*t*
	OF	18	32	24.0	23.50		ns	4	9	7.0	6.7		ns	10	12	10.5	10.8		ns	3	13	5	6.0		ns

Supplementary Table S2. Results on statistical differences between habitats, species and sites in the concentrations of pesticides quantified by individual.

The abbrevations of factor levels are: conventional farming “CF”, organic farming “OF”, wood mouse *Apodemus sylvaticus* “Apsy”, and shrews *Crocidura russula* “Crru”. The statistical tests performed are referred to as ANOVA “AOV”, Kruskal-Wallis “KW”, t-test “*t*”, and Wilcoxon-Mann-Whitney “MN”. Statical significance is indicated with the following signs: “ns” non significant *p-value* > 0.05 (in case the p-value was close to significance the exact *p-value* is provided), * for 0.05 ≥ *p-value* > 0.01, ** for 0.01 ≥ *p-value* > 0.001, *** *p-value* < 0.001. Significant differences between levels are indicated by uppercase letters, levels sharing the same letter did not differed statistically.

Dataset	Factors levels	Concentrations of Fungicides						Concentrations of Herbicides						Concentrations of Insecticides					
		Min	Max	Med	Mean		Test	Min	Max	Med	Mean		Test	Min	Max	Med	Mean		Test
**BRPs**																			
Shrews from ZAPVS in three	**Habitat**																		
habitats	Cereal	0.048	1.50	0.344	0.440		KW	**2.12**	**12.3**	**2.72**	**4.00**	a	**KW**	**7.57**	**60.5**	**13.2**	**16.2**	a	**KW**
(*n*=63)	Grassland	0.051	0.703	0.097	0.168		ns	**1.73**	**4.91**	**3.27**	**3.19**	a	***	**6.41**	**11.5**	**8.94**	**8.71**	a	***
	Hedgerow	0.000	3.62	0.203	0.697			**0.471**	**9.33**	**1.04**	**1.51**	b		**10.8**	**128**	**24.1**	**33.7**	b	
	**Farming**																		
	CF	0.000	2.89	0.232	0.479		MN	**0.535**	**12.3**	**2.55**	**2.95**	a	**MN**	6.41	128	16.5	22.0		MN
	OF	0.016	3.62	0.143	0.753		ns	**0.471**	**4.44**	**1.09**	**1.44**	b	**	7.35	114	19.2	33.0		ns
Shrews and wood mice	**Species**																		
from ZAPVS in hedgerows	Apsy	**0.037**	**0.543**	**0.087**	**0.117**	a	**MN**	**4.56**	**47.2**	**12.2**	**18.1**	a	**MN**	7.95	120	16.0	26.8		MN
(*n*=28)	Crru	**0.000**	**2.77**	**0.473**	**0.911**	b	***	**0.471**	**4.83**	**1.05**	**1.44**	b	***	12.9	128	24.4	33.8		ns
	**Farming**																		
	CF	0.000	2.03	0.141	0.375		MN	0.798	47.2	4.83	10.8		MN	7.95	128	24.2	34.8		MN
	OF	0.037	2.77	0.266	0.861		ns	0.471	29.2	3.15	7.12		ns	8.15	40.9	17.4	19.1		ns
Wild mice from the two	**Zone**																		
zones in hedgerows (*n*=30)	ZAAJ	**0.000**	**0.193**	**0.001**	**0.021**	a	**MN**	**0.511**	**7.77**	**1.03**	**2.10**	a	**MN**	8.18	28.4	16.6	17.2		MN
	ZAPVS	**0.037**	**0.543**	**0.087**	**0.117**	b	***	**4.56**	**47.2**	**12.2**	**18.1**	b	***	7.95	120	16.0	26.8		ns
	**Farming**																		
	CF	0	0.543	0.048	0.081		MN	0.511	47.2	4.95	11.1		MN	7.95	120	16.29	24.2		MN
	OF	0	0.128	0.003	0.035		*p* = 0.094	0.596	29.2	3.81	6.41		ns	8.15	22.9	16.6	16.6		ns
**CUPs**																			
Shrews from ZAPVS in three	**Habitat**																		
habitats	Cereal	4.64	360	40.1	64.6		KW	**7.37**	**293**	**15.3**	**50.6**	ab	**KW**	**4.21**	**75.2**	**16.1**	**22.9**	a	**KW**
(*n*=63)	Grassland	1.91	181	4.63	38.6		ns	**6.14**	**30.2**	**11.5**	**14.6**	a	*	**2.40**	**9.85**	**4.33**	**5.08**	b	**
	Hedgerow	3.44	249	42.0	65.5			**5.26**	**989**	**38.7**	**100**	b		**3.29**	**62.1**	**13.1**	**17.6**	a	
	**Farming**																		
	CF	1.91	360	41.8	65.3		MN	6.14	989	22.9	80.2		MN	2.40	75.2	13.9	19.0		MN
	OF	3.44	212	13.9	53.1		ns	5.26	439	15.6	52.9		ns	3.29	62.1	6.79	15.2		*p* = 0.058
Shrews and wood mice	**Species**																		
from ZAPVS in hedgerows	Apsy	1.16	451	33.4	85.8		MN	12.9	197	26.7	45.7		MN	**1.23**	**99.0**	**3.27**	**14.4**	a	**MN**
(*n*=28)	Crru	3.84	249	56.0	81.7		ns	7.60	439	51.2	117		ns	**3.66**	**62.1**	**18.5**	**22.4**	b	**
	**Farming**																		
	CF	1.16	451	40.2	90.6		MN	8.72	364	47.6	83.3		MN	1.33	99.0	11.8	19.2		MN
	OF	5.33	202	34.2	66.7		ns	7.60	439	20.0	77.8		ns	1.23	62.1	4.22	16.6		ns
Wild mice from the two	**Zone**																		
zones in hedgerows (*n*=30)	ZAAJ	**0.527**	**16.1**	**1.20**	**2.52**	a	**MN**	**4.61**	**47.2**	**6.37**	**8.96**	a	**MN**	**0.430**	**6.73**	**0.989**	**1.60**	a	**MN**
	ZAPVS	**1.16**	**451**	**33.4**	**85.8**	b	***	**12.9**	**197**	**26.7**	**45.7**	b	***	**1.23**	**99.0**	**3.27**	**14.4**	b	***
	**Farming**																		
	CF	0.526	451	7.32	57.0		MN	4.61	197	13.3	32.1		MN	0.575	99.0	2.33	10.3		MN
	OF	0.729	45.0	1.41	10.2		ns	5.06	52.0	6.63	14.0		ns	0.430	9.66	1.22	2.24		ns

Supplementary Table S3. Extract of sales of pesticides in 2016 in Deux-Sèvres (location of site ZAPVS where most of small mammals have been captured) ordered according to the quantity sold.

Compounds are ranked by decreasing quantities. The compounds screened in the study are highlighted in yellow.

Rank	Compound	Rank	Compound	Rank	Compound
1	glyphosate	101	tetraconazole	201	acetic acide
2	fosetyl-aluminium	102	cymoxanil	202	ipconazole
3	prosulfocarb	103	quinoxyfen	203	acequinocyl
4	sulfur for spraying (micronized)	104	vaseline oil	204	desmedipham
5	mancozeb	105	clethodim	205	fenbuconazole
6	folpel	106	cloquintocet-mexyl	206	flumioxazin
7	s-metolachlor	107	fenpropidin	207	spirotetramat
8	potassium phosphonate	108	pelargonic acid	208	methanol
9	copper from copper sulphate	109	silthiofam	209	1-dodecanol
10	metiram	110	thiamethoxam	210	rosin resin
11	chlortoluron	111	mefenpyr-diethyl	211	triticonazole
12	pendimethalin	112	pyrimethanil	212	fenoxaprop-p-ethyl
13	chlorothalonil	113	trinexapac-ethyl	213	alpha-naphthaleneacetic acid
14	aclonifen	114	beflubutamid	214	bupirimate
15	disodium phosphonate	115	picolinafen	215	iprodione
16	dimethenamid-p (dmta-p)	116	metamitron	216	emamectin benzoate
17	metaldehyde	117	imazamox	217	esfenvalerate
18	copper from copper hydroxide	118	kresoxim-methyl	218	triflusulfuron-methyl
19	isoproturon	119	meptyldinocap	219	metobromuron
20	prothioconazole	120	carboxine	220	barium nitrate
21	copper from cuprous oxide	121	potassium bicarbonate	221	sulfosulfuron
22	chlormequat chlorure	122	mecoprop-p (mcpp-p)	222	*Bacillus thuringiensis* ssp *kurstaki*
23	propyzamide	123	lambda-cyhalothrine	223	thiabendazole
24	captane	124	dimethomorph	224	copper tricupric and tricalcium sulfate
25	tebuconazole	125	clodinafop-propargyl	225	iprovalicarb
26	metazachlor	126	diquat	226	anthraquinone
27	epoxiconazole	127	sedaxane	227	pyrimiphos-methyl
28	prochloraz	128	tritosulfuron	228	benalaxyl
29	napropamide	129	decane-1-ol	229	octanoica acid
30	iron sulphate	130	bifenox	230	powdered sulphur
31	2,4-mcpa	131	ventilated sulphur	231	chloridazon
32	diflufenicanil	132	metalaxyl-m	232	oxyfluorfene
33	imidaclopride	133	rapeseed oil	233	bromuconazole
34	spiroxamine	134	mepanipyrim	234	acrinathrin
35	flurochloridone	135	ferric sulphate	235	flupyrsulfuron-methyl
36	dichlorprop-p	136	tembotrione	236	*Bacillus subtilis*
37	dimethachlore	137	prosulfuron	237	abamectin
38	2,4-d	138	metribuzin	238	proquinazid
39	flurtamone	139	prohexadione-calcium	239	pencycuron
40	metrafenone	140	phosmet	240	decanoic acid
41	boscalid	141	picoxystrobin	241	1-tetradecanol
42	flufenacet	142	tau-fluvalinate	242	*Bacillus pumilus* strain qst 2808
43	soufre	143	sulcotrione	243	mandipropamid
44	ametoctradine	144	tefluthrin	244	linuron
45	cyprodinyl	145	clopyralid	245	oil-resin
46	dicamba	146	2,4-db	246	castor oi
47	thirame	147	metsulfuron-methyl	247	polybuten
48	dithianon	148	pyroxsulam	248	vegetable oil
49	fluxapyroxad	149	propoxycarbazone sodium	249	spinosad
50	pyraclostrobin	150	mesosulfuron-methyl	250	clothianidine
51	quinmerac	151	alphamethrin	251	tebufenpyrad
52	mepiquat-chloride	152	florasulam	252	spirodiclofen
53	chlorpyriphos-methyl	153	ethofumesate	253	flazasulfuron
54	azoxystrobin	154	fluazifop-p-butyl	254	chlorpropham
55	mesotrione	155	cyflufenamid	255	benzovindiflupyr
56	fish oil	156	methiocarb	256	imazaquin
57	propiconazole	157	fenhexamid	257	oryzalin
58	ethephon	158	isoxadifen-ethyl	258	penoxsulam
59	fenpropimorph	159	2,4-mcpb	259	beeswax
60	cypermethrin	160	deltamethrin	260	ioxynil
61	Bromoxynil-octanoate	161	glufosinate ammonium	261	polyisobuten
62	isoxaflutole	162	tribenuron-methyl	262	alpha-naphtyl-acetamid

63	choline chloride
64	metconazole
65	zoxamide
66	cyproconazole
67	chlorpyriphos-ethyl
68	fluroxypyr
69	fluopyram
70	bentazone
71	cyazofamide
72	fluopicolide
73	trifloxystrobin
74	ferric phosphate
75	bixafen
76	thiophanate-methyl
77	sublimed sulphur
78	dodine
79	pinoxaden
80	cyprosulfamide
81	fluoxastrobin
82	mecoprop (mcpp)
83	nicosulfuron
84	pethoxamide
85	benalaxyl-m
86	pepper
87	fludioxonil
88	thiaclopride
89	triallate
90	cycloxydim
91	pyrimicarb
92	paraffinic mineral oil
93	difenoconazole
94	triclopyr
95	bromoxynil
96	isoxaben
97	thiencarbazone-methyl
98	clomazone
99	benoxacor
100	chlorantraniliprole
163	dimoxystrobin
164	metiram-zinc
165	aminopyralid
166	clopyralid
167	diflubenzuron
168	flonicamid
169	iodosulfuron-methyl-sodium
170	slaked lime
171	quizalofop-p-ethyl
172	propaquizafop
173	zetacypermethrin
174	thifensulfuron-methyl
175	cresyl
176	carfentrazone-ethyl
177	foramsulfuron
178	e8,e10-dodecadiene-1-ol
179	indoxacarb
180	lenacil
181	essential oil of sweet orange
182	6-benzyladenin
183	carbetamide
184	phenmedipham
185	benfluralin
186	amidosulfuron
187	pine tars
188	paclobutrazol
189	ethyl alcohol
190	pyridate
191	piperonyl butoxide
192	maleic hydrazide
193	Cationic polymer carbonyl sulfide
194	fenazaquin
195	etofenprox
196	propamocarb
197	laminarine
198	acetampride
199	fenoxycarb
200	copper oxychloride
263	tribasic copper sulphate
264	hexythiazox
265	pyraflufen-ethyl
266	gibberellic acid
267	rimsulfuron
268	pyrethrins
269	hymexazol
270	Diclofop-methyl
271	benthiavalicarb
272	amitrole
273	Ammonium thiocyanate
274	miscellaneous fertilisers
275	myclobutanil
276	oxadiazon
277	rotenone
278	z-8-dodecenyl acetate
279	spinetoram
280	sodium hydroxide
281	gibberellins (a4+a7)
282	b-indole butyric acid
283	natural pyrethrum
284	propineb
285	sodium hypochlorite
286	acibenzolar-s-methyl
287	*Bacillus thuringiensis* serotype 3a 3b
288	e-8-dodecenyl acetate
289	dimethyl polysiloxane
290	picloram
291	triadimenol
292	cresol
293	(z)-8-dodecenol
294	chlorophacinone

Supplementary Methods. Molecular identification of small mammal species

Total genomic DNA was extracted from 10-25mg of ear tissue using the DNeasy Blood and Tissue kit (Qiagen, Courtaboeuf, France) according to the manufacturer's recommendations (pp 28-30, version 07/2006). Each sample was processed independently in an automated manner using the QIAcube robot (Qiagen). DNA concentration was then measured using a NanoVue Plus spectrophotometer (Biochrom). DNA extracts were stored at −20 °C until DNA amplification.

All DNA extracts were then amplified for an approximatively 900 bp-long fragment (excluding primers) of the *cytochrome b* gene. We used primers CytB Uni fw 5' – TCATCMTGATGAAAYTTYGG – 3' and CytB Uni rev 5' – ACTGGYTGDCCBCCRATTCA – 3' published in Schlegel et al. (2011). Amplifications were performed in 50µL reactions containing 1× HotStart Taq Master Mix (Qiagen), 0.32µM of each primer, 0.5ng/µL of bovine albumin serum, 0.75mM of $MgCl_2$, and 5µL of DNA extract (10-70 ng/µL). The PCR program consisted in an activation step of 15mn at 95°C, followed by 40 cycles of denaturation at 94°C during 30s, annealing at 53°C during 45s, and extension at 72°C during 1mn. A final extension was performed at 72°C during 10mn. Amplification reactions were performed using an Eppendorf Mastercycler DNA Engine.

The PCR products were separated and visualized using the QIAxcel device and a QIAxcel DNA Screening kit (Qiagen). The amplified products were purified using the Illustra GFX PCR DNA and Gel Band Purification Kit (GE Healthcare) according to the manufacturer's instructions. Direct sequencing of the PCR products was performed with an automated sequencer (Applied Biosystems Seqstudio Genetic Analyzer). All the samples were sequenced with two primers employed for the PCR reactions. The DNA sequences obtained were submitted to GenBank with the Blast algorithm (http://blast.ncbi.nlm.nih.gov/Blast.cgi). A query sequence was then assigned to a given species when identity and coverage matches the database sequence with a threshold of at least 99% for only one species.

References

Schlegel, M., Ali, H. S., Stieger, N., Groschup, M. H., Wolf, R., & Ulrich, R. G. (2012). Molecular identification of small mammal species using novel cytochrome B gene-derived degenerated primers. *Biochemical genetics*, *50*(5-6), 440-447.

Supplementary Figure S1. Relationships between A) number of detection in hair of small mammals or B) number of quantification above 10 ng/g in hair of small mammals and the quantity of each corresponding pesticide sold in 2016 in Deux-Sèvres (location of site ZAPVS where most of small mammals have been captured) with results of non-parametric tests of correlation.

The metabolites of pyrethrinoids were not included in the analyses since they could not be related to specific corresponding pesticides.

A)

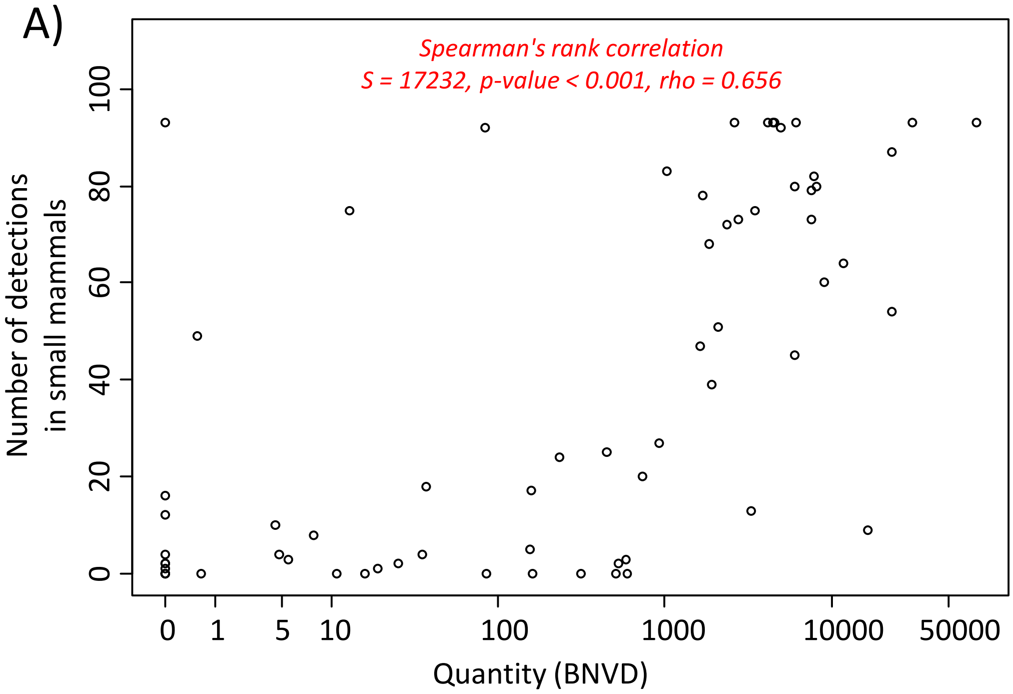

B)

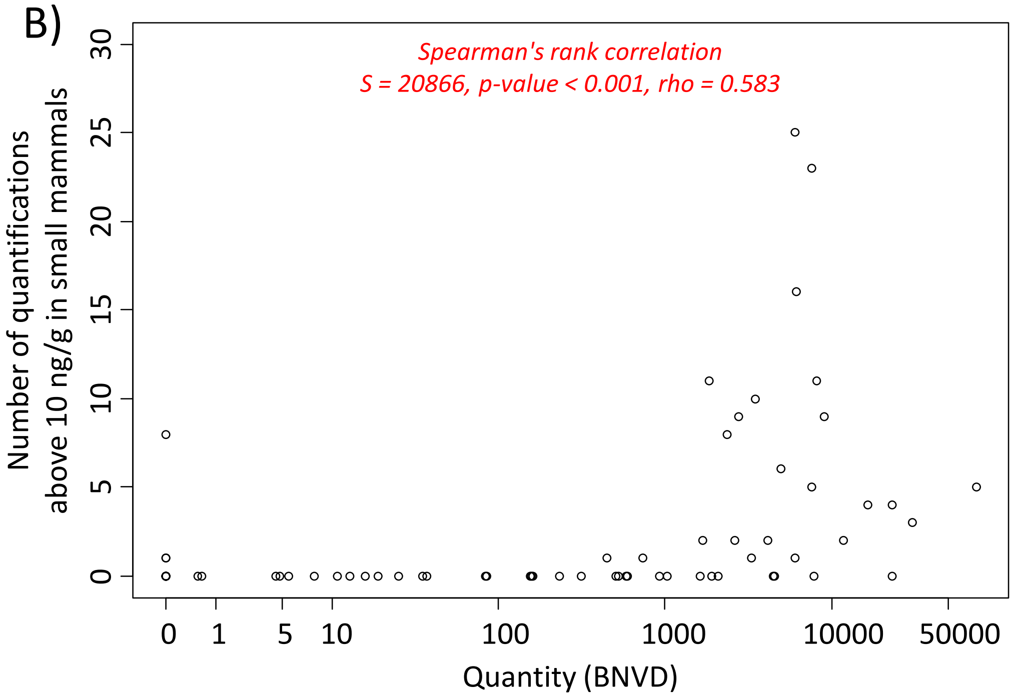

Supplementary Figure S2. Correspondence between parent organophosphorous pesticides and their metabolites.

Chemicals analyzed in this study are highlighted by green squares. Parent pesticides tagged with "" were currently used (CUPs) at the time of the study, and those tagged with "°" were banned (BRPs). The abbreviation "DAPs" means dialkyl phosphates.*

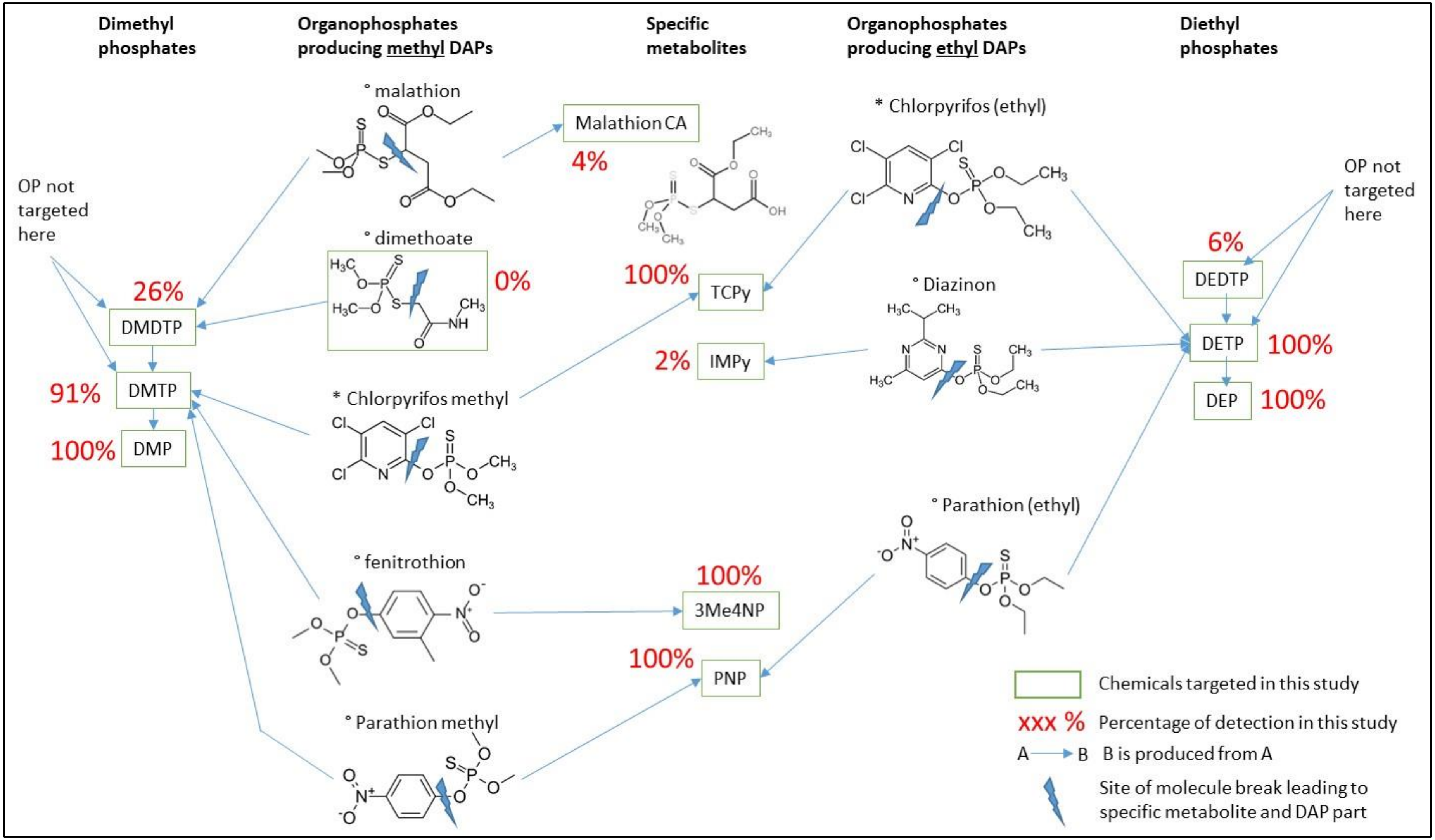

Supplement: Supplementary file 2 — Supplementary Information 2. [file 41598_2022_19959_MOESM2_ESM.pdf]
